# Fecal Microbiota Transplantation Is Associated with Better Survival Compared to Standard of Care in Severe Alcoholic Hepatitis Not Responding to Corticosteroids: A Systematic Review and Meta-Analysis

**DOI:** 10.3390/jcm15135131

**Published:** 2026-07-01

**Authors:** Jakub Hoferica, Bettina Csilla Budai, Eszter Ágnes Szalai, Ádám Zolcsák, Marie Anne Engh, Katalin Lenti, Péter Hegyi, Jun Yu, Péter Jenő Hegyi, Peter Banovcin

**Affiliations:** 1 Centre for Translational Medicine, Semmelweis University, 1085 Budapest, Hungary; hoferica5@uniba.sk (J.H.);; 2 Jessenius Faculty of Medicine in Martin, Comenius University, 03601 Martin, Slovakia; 3 Institute of Pancreatic Diseases, Semmelweis University, 1083 Budapest, Hungary; 4 Department of Restorative Dentistry and Endodontics, Semmelweis University, 1088 Budapest, Hungary; 5 Department of Biophysics and Radiation Biology, Semmelweis University, 1094 Budapest, Hungary; 6 Department of Morphology and Physiology, Faculty of Health Sciences, Semmelweis University, 1088 Budapest, Hungary; 7 Institute for Translational Medicine, Medical School, University of Pécs, 7624 Pécs, Hungary; 8 Translational Pancreatology Research Group, Interdisciplinary Centre of Excellence for Research Development and Innovation University of Szeged, 6720 Szeged, Hungary; 9 Institute of Digestive Disease and Department of Medicine and Therapeutics, State Key Laboratory on Digestive Disease, Li Ka Shing Institute of Health Science, Chinese University of Hong Kong, Hong Kong, China

**Keywords:** gut microbiota, gut–liver axis, alcoholic hepatitis, alcohol-related liver disease, fecal microbiota transplantation

## Abstract

**Background**: Alcohol-related liver disease (ALD) affects 4.8% of the global population. Among these patients, between 13.4% and 19.6% suffer from alcoholic hepatitis (AH), which, in its severe form, is associated with significant short- and long-term mortality. Current therapeutic options are limited, offering only modest short-term survival benefits. Recent studies suggest that microbiota-based therapies may offer a novel therapeutic opportunity for patients with ALD. **Methods**: Databases including Embase, Medline, and CENTRAL were searched until 4 February 2026. The pre-registered protocol on PROSPERO (CRD42023467455) was followed without deviation. Studies comparing adult patients with ALD, treated with fecal microbiota transplantation (FMT) or standard of care (SOC), were included. Outcomes investigated included overall survival, alcoholic recidivism, adverse events (AEs), and disease severity scores. Risk of bias (ROB) was assessed using the ROBINS-I and ROB 2 tools. Hazard ratios (HR) were calculated for FMT versus SOC groups. **Results**: Overall, 10 studies were eligible for inclusion, with 339 patients eligible for synthesis. In these patients, FMT was associated with significantly improved overall survival compared to the SOC, with an HR of 0.50 (95% confidence interval (CI): 0.35–0.72; *p* = 0.0002). When comparing FMT with pentoxifylline, the HR was 0.45 (95% CI: 0.21–0.96; *p* = 0.0345), and when FMT was compared with nutritional support alone, the HR was 0.36 (95% CI: 0.19–0.66; *p* = 0.0001). But FMT did not reach statistical significance when compared to glucocorticoids. ROB analysis showed a moderate to high risk of bias, and the overall certainty of evidence was low. **Discussion**: FMT is a promising therapeutic option for improving short- and medium-term survival in patients with severe alcoholic hepatitis (SAH), particularly in those who are ineligible or unresponsive to corticosteroid therapy. However, given the risk of bias and low certainty of evidence, clinical significance remains uncertain. Confirmation in well-designed studies is needed.

## 1. Introduction

Over the past decades, global alcohol consumption has been increasing. In 2017, 20% of adults were heavy episodic drinkers, and this prevalence is projected to rise to 23% by 2030 [1]. Alcohol-related liver disease (ALD) is currently affecting 4.8% of the global population. Among these patients, from 13.4% to 19.6% develop alcoholic hepatitis (AH) [2]. This syndrome is associated with significant short-term and medium-term mortality rates, reaching 20% and 44%, respectively, in severe cases. Consequently, ALD imposes a significant burden on healthcare systems worldwide [3].

Currently, therapeutic options for severe AH remain limited to supportive care, glucocorticoids, nutritional, and vitamin supplementation [4]. Glucocorticoids are considered to be the hallmark of pharmacological treatment for severe alcoholic hepatitis (SAH), but their use is restricted to highly selected patients. Furthermore, the overall effect of glucocorticoids on mortality is relatively low and observed only in the short term [5]. Several complications and contraindications related to corticosteroid therapy limit their broad application in AH. In recent real-world studies, 41% of patients with SAH were ineligible for glucocorticoids treatment, and 29% were non-responders, resulting in the majority of patients not benefiting from this therapy [6]. Despite decades-long exploration of several novel treatment options, none have been proven effective. This underscores the need for innovative treatments, particularly for non-responders to glucocorticoid therapy, as highlighted in the American Association for the Study of Liver Diseases guidelines [7].

In recent years, a better understanding of the intestinal microbiota, the gut–liver axis, has led to novel insights into pathophysiological pathways behind the development and progression of AH [8]. These insights, together with numerous animal models and human studies suggest that alterations in gut microbiota composition may play a crucial role in disease severity and outcomes [9]. Additionally, organizations such as the American Association for the Study of Liver Diseases, the American College of Gastroenterology, and the European Association for the Study of the Liver have recognized the potential of microbiota-based therapies for patients with ALD and AH [4,7,8]. However, the level of evidence remains low; thus, strong recommendations have not yet been provided in the guidelines.

We aim to evaluate and synthesize the evidence available in the current literature on the application of fecal microbiota transplantation (FMT) in patients with ALD, especially in those with SAH.

## 2. Materials and Methods

The Cochrane Handbook was followed for standards and methods [10] (see Supplementary Table S1), and PRISMA [11] for reporting. The protocol was registered in advance on Prospero with the code ID CRD42023467455, and we fully adhered to it.

Eligibility criteria.

Studies were eligible for inclusion if they involved (P) adult patients diagnosed with ALD, including alcoholic fatty liver disease, AH, or alcoholic cirrhosis. When comparing (I) FMT to (C) the standard of care (SOC), the primary outcomes were overall survival, alcoholic recidivism, and AEs. Studies were excluded if they were case reports or case series as their study design.

Information sources.

On September 27, 2023, a systematic search was conducted with the same search key and without language or other restrictions, using Embase, Medline, and Cochrane Central Register of Controlled Trials (CENTRAL). The search was subsequently updated on February 4, 2026. The references of the included studies were systematically searched using citation chaser(v0.0.3) [12].

Search strategy.

The search strategy was designed using a search key consisting of two main domains: the first focused on defining ALD, and the second on the FMT procedure. For details on search key, see the Supplementary Materials.

Selection process.

Screening and selection were performed entirely by two review authors (JH and BB) independently in a stepwise manner. Automatic and manual duplicate removal was performed in EndNote (v20, Clarivate Analytics, Philadelphia, PA, USA). Title and abstract selection was performed in Rayyan [13]. The level of agreement at each step was assessed using Cohen’s kappa coefficient. In case of disagreement during the selection, a third independent party was assigned to resolve it (PB).

Data collection process.

Two independent authors (JH and BB) extracted data from eligible articles. To minimize the chance of error, we collected data separately with subsequent comparisons of extracted data. A predefined Excel table (Office 365, Microsoft, Redmond, WA, USA) was used for data collection. A third independent party (PB) resolved all disagreements.

Data items.

The following data were extracted: first author; year of publication; study location and period; study population size, age, disease severity scores, diagnostic criteria, FMT administration method, dosage and duration; SOC definition; survival time; microbiota information; alcohol use recurrence; and AEs, including rates of infections, sepsis, organ failure, ascites, hepatic encephalopathy, and disease severity scores.

Study risk of bias assessment (ROB) and certainty of evidence.

The ROBINS-I [14] tool was used for non-randomized studies, while the ROB 2 tool [15] was used for randomized controlled trials (RCT). The assessment was performed by two independent investigators (JH and BB). All disagreements were resolved by an independent third party (PB). Results were visualized using the robvis tool [16]. The level of evidence was assessed by GRADEpro [17].

Synthesis methods.

As we assumed considerable heterogeneity among the underlying population of the reported study results due to the nature of the examined question, random effects models were used in a frequentist framework. The minimum number of studies required for analysis was 3. In the case of any overlapping population, the study with large samples size or longer follow-up was used.

For time-to-event data, we used hazard ratios (HRs) with a 95% confidence interval as the main measure of effect between the two groups: FMT and SOC. We calculated HRs as FMT vs. SOC groups. To calculate the pooled HR, we used two methods: (1) a classical random-effects meta-analysis method (hereafter referred to as the ‘classical’ method) based on calculated study HRs, and (2) an individual patient data (IPD)-based random-effects Cox hazard model with Gaussian random effects (hereafter referred to as ‘IPD-based’). Additionally, as a secondary measure, we estimated survival probabilities at specific time points and pooled them with a 3-level model (“multilevel”) to indirectly compare survival in the groups. An estimation for distribution-free pooled survival curves was also performed using the method implemented by Pandey [18] (“curve estimate”). An additional analysis was performed using the IPD-based model to compare the HR in the experimental (FMT) and separately in the three different control groups nutritional support pentoxifylline and glucocorticoids.

When articles did not report HR and used varying outcome measures for time-to-event data, but provided Kaplan–Meier curves, we derived IPD estimates from these plots. We used the free WebPlotDigitizer(v5.2) tool to extract values from the plots [19]. We compared the extracted and calculated data with the published data where possible.

Results were considered statistically significant if the pooled CI does not contain the null value. We summarized the findings in tables, in forest plots, Kaplan–Meier plots (using Kaplan–Meier estimates), and estimated survival curves. Where appropriate, between-study heterogeneity was described by the between-study variance (τ2) and also I2 statistics based on the classical method, and the 3-level method. For IPD-based results, we reported variance of the random effects. Small study publication bias in the classical and multilevel methods was assessed by visual inspection of funnel-plots and calculating Egger’s test *p*-value [20] using a 10% significance level. However, we assumed possible small study bias based on the *p*-value if the study number was at least 10. Potential outlier publications were explored using different influence measures and plots following the recommendation of Harrer et al. [21].

All statistical analyses were calculated by R software (v4.4.2, Foundation for Statistical Computing, Vienna, Austria) [22] using the meta [23,24] (v7.0.0) package for basic meta-analysis calculations and plots, and the dmetar [25] (v0.1.0) package for additional influential analysis calculations and plots for the classical method. The package metafor [26] (v4.4.0) was used for the multilevel model. The packages survival [27,28] (v3.7.0), survminer [29] (v0.4.9), and coxme [30] (v2.2.20) were used for IPD-based calculations. MetaSurvival [18] (v0.1.0) package was used with the ecurve estimation method. For additional details on calculations, data synthesis, publication bias assessment and influential analyses; see the detailed description [31,32,33,34,35,36,37,38].

During the preparation of this review, the author used AI-based language tools [Claude (model: Claude Sonnet 4.5, Anthropic, San Francisco, CA, USA)] and [ChatGPT (model: GPT-4o, OpenAI, San Francisco, CA, USA)] solely to proofread, check grammar, spelling, punctuation, and sentence structure.

## 3. Results

### 3.1. Search and Selection

**Figure 1:**
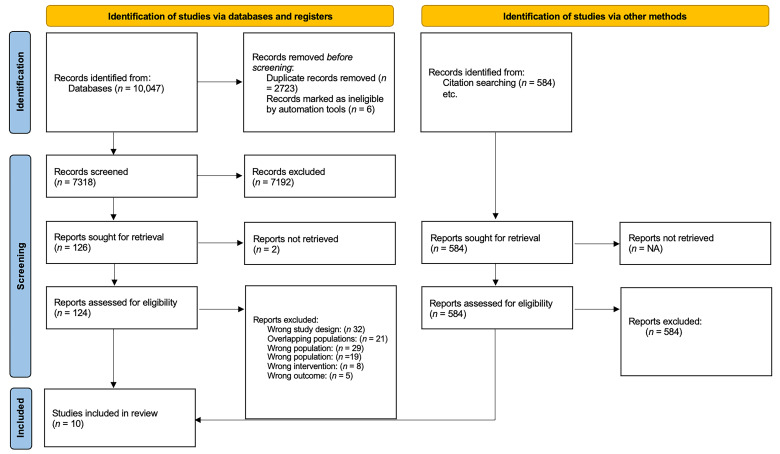
PRISMA 2020 flowchart representing the study’s updated selection process.

Altogether, 6512 studies were identified. After duplicate removal, 4852 studies remained for title and abstract screening, of which 97 were selected for full-text review (Cohen’s kappa = 0.89). Finally, 10 studies [39,40,41,42,43,44,45,46,47,48] were deemed eligible for data extraction (Cohen’s kappa = 0.91). The systematic search of references of included studies did not yield any additional findings. The updated search yielded 3535 new records, of which one additional record from a previously published abstract was identified [49]. See Figure 1 for updated search.

### 3.2. Basic Characteristics of Included Studies

A total of 494 patients were included in this systematic review, of whom 339 were eligible for synthesis. Of the ten studies, nine reported on SAH, one on cirrhosis [40], and one on SAH with acute-on-chronic liver failure [42]. None reported the use of FMT in alcohol-related steatosis. Eight studies were conducted in India, one in the USA, and one in Slovakia. FMT was administered via nasoduodenal tube in five studies. One study reported the use of nasojejunal tube, and another used enema. The reported dose of FMT ranged from 27 g to 30 g or was 100 mL administered once daily. The duration of FMT application was seven days in five studies and six days in one study. Interestingly, Sharma et al. [48] reported a single dose of FMT with a similar effect size to other studies. Studies investigating SAH noted high clinical heterogeneity in SOC. FMT donors were either unrelated healthy volunteers or healthy related family members. For details, see Table 1.

### 3.3. FMT Treatment Exhibits Significantly Better Overall Survival Compared to Standard of Care in Severe Alcoholic Hepatitis

**Figure 2:**
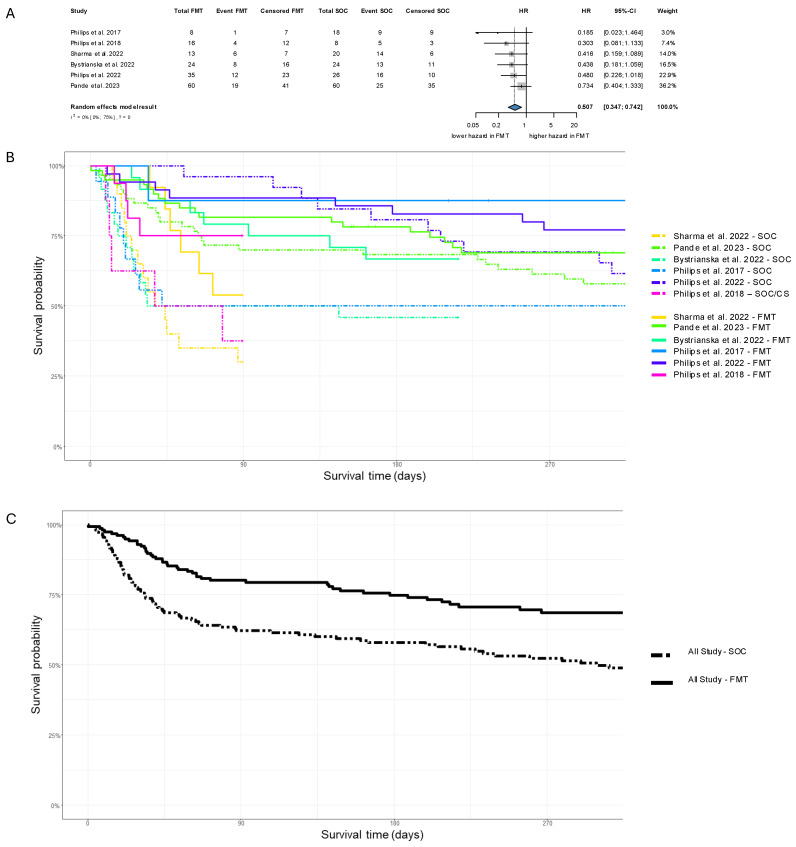
Survival analysis based on IPD (**A**) Forest plot representing pooled hazard ratio for survival comparing SOC vs. FMT in SAH. (**B**) Kaplan–Meier curve showing IPD survival data in SOC vs. FMT. (**C**) Pooled Kaplan–Meier curve for all studies. IPD: individual patient data; SAH: severe alcoholic hepatitis; SOC: standard of care; FMT: fecal microbiota transplantation; HR: hazard ratio; CI: confidence interval. [40,42,44,46,47,48].

In six studies [40,42,44,46,47,48] comprising 339 cases, FMT was associated with better survival compared to SOC. An HR of 0.5075 (95% CI 0.3469; 0.7423) was based on pooling data from recalculated HRs, and an HR of 0.50 (95% CI 0.35; 0.72), *p* = 0.0002, was based on a Random Effects Model on IPD. See Figure 2 and Supplementary Figures S1 and S2.

In a study by Philips et al., 2018 [47] multiple SOC arms (pentoxifylline, nutritional support, glucocorticoids) were used. To avoid overlaps with the FMT arm, only the glucocorticoids arm was pooled. The models with pentoxifylline and nutritional support arms can be found in Supplementary Figures S3–S24. Furthermore, estimates of IPD for FMT arm in the study by Philips et al. (2022) [44] were nearly identical to the study of Philips et al. (2022) [45]; therefore, only the study with the large sample size was used to avoid any potential overlapping of populations.

In IPD pairwise comparisons for all treatments, FMT demonstrated improved outcomes compared to the pentoxifylline, and nutritional support arms. HRs were 0.65 (95% CI 0.33; 1.28), *p* = 0.3642 for glucocorticoids; 0.45 (95% CI 0.21; 0.96), *p* = 0.0345 for pentoxifylline; and 0.36 (95% CI 0.19; 0.66), *p* = 0.0001 for nutritional support, respectively, see Supplementary Table S2.

The survival probabilities at 28, 90, and 180 days were higher for FMT than for SOC. For SOC, the survival probability was 79% at 28 days (95% CI: from 51 to 93%), it was 64% at 90 days (95% CI: from 33 to 86%), and 62% at 180 days (95% CI: from 24 to 89%). In contrast, for FMT, the survival probability was 95% at 28 days (95% CI: from 85 to 99%), it was 80% at 90 days (95% CI: from 47 to 95%), and it remained at 80% at 180 days (95% CI: from 42 to 95%). For more details, including forest plots, leave-one-out (Loo) analyses, and models utilizing the Philips et al. (2018) [47], pentoxifylline, and nutritional support arms for survival probabilities at 28, 90, and 180 days, see Supplementary Figures S13–S26.

The Loo sensitivity analysis indicates that the study by Pande et al. [42] is an influential study, with an effect size of 0.41 (95% CI: from 0.26 to 0.65). Nonetheless, the overlapping confidence intervals for the Loo estimates suggest that the meta-analytic conclusions remain robust and are not driven solely by any single study.

### 3.4. FMT Application Is Associated with Lasting Changes in the Gut Microbiota of Patients with SAH

Six studies [42,43,44,45,46,47] included microbiota analysis. All studies used 16S rRNA sequencing, focusing on the V3–V4 region. Three studies reported differences in baseline gut microbiome between SAH/FMT recipients and FMT donors or healthy controls. Pande et al. [42] observed a 23% variation in the first two principal components of bacterial taxa between donors and SAH patients. Philips et al. (2018) [47] observed higher Proteobacteria and Lentisphaerae in controls, whereas Firmicutes and Actinobacteria were higher in SAH. In contrast, Philips et al. (2017) [46] found no significant donor-recipient differences.

The change in gut microbiota followed by FMT was reported in five studies [42,44,45,46,47]. FMT increased α-diversity and shifted the microbiota community toward a donor-like profile. At the family level, increases were noted in Veillonellaceae, Lachnospiraceae, Bifidobacteriaceae, Bacteroidaceae, Porphyromonadaceae, and Tannerellaceae. Conversely, decreases were consistently reported for Enterobacteriaceae, Streptococcaceae, Campylobacteraceae, and Desulfovibrionaceae and, in some reports, Moraxellaceae, Weissellaceae, Leuconostocaceae, and Aerococcaceae changes were consistent with greater microbial stability and a healthier microbiota profile.

Survival analyses suggested favorable associations with Succinivibrionaceae, Lachnospiraceae, Veillonellaceae, and Prevotellaceae. In contrast, higher abundance of Enterobacteriaceae and Mycoplasmataceae was observed in non-survivors. See Supplementary Table S3.

### 3.5. Secondary Outcomes Such as Recurrence of Alcohol Use and Severity Scores Showed No Major Difference Between FMT and SOC at Baseline

**Figure 3:**
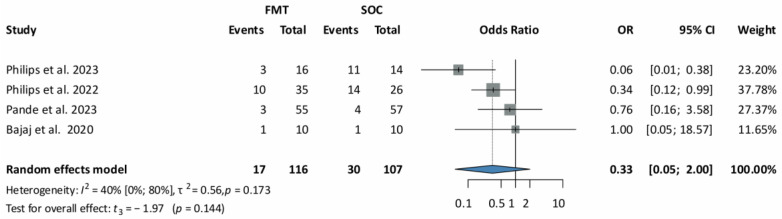
The odds ratio for the recurrence of alcohol use after treatment. OR: odds ratio; CI: confidence interval, SOC: standard of care; FMT: fecal microbiota transplantation [40,42,43,44].

On the basis of four [40,42,43,44] articles and 223 cases, the recurrence of alcohol use was lower in the FMT arm, with an odds ratio of 0.33 (95%CI 0.05 to 2) *p* = 0.144; I2 = 40% (95%CI 0 to 80), not reaching clinical or mathematical significance. See Figure 3.

Disease severity scores (DSC), such as the Maddrey’s Discriminant Function (MDF), Child-Pugh Score for Cirrhosis (CPT), and Model for End-Stage Liver Disease (MELD), were evaluated in four, five, and seven studies, respectively. Overall, the mean difference between baseline FMT and SOC was comparable for MDF and MELD, whereas the CPT score showed an overall slightly higher score in the FMT arm. Notable outliers for MELD were observed by Philips et al. (2018) [47] for the pentoxifylline arm, with a mean difference of −2.1 favoring FMT, and by Philips et al. (2017) [46], with a mean difference of 4 points favoring SOC. For CPT, Philips et al. (2017) [46] once again stood out as a notable outlier, reporting a mean difference of 2.3 points in favor of SOC. For MDF, Philips et al. (2018) [47] showed a mean difference greater than 3 points across all three arms, favoring FMT. Despite these outliers, the majority of included studies showed similar DSC in the included population in the study. See Supplementary Figures S25–S27 and Supplementary Table S4. The changes in DSC before and after FMT treatment were reported in four studies [40,42,46,48] of ALD and in three studies [40,42,46,48] of SAH populations. The reported decreases of DSC ranged from –4.20 to –18.70 for MELD, from –1.80 to –6.80 for CPT, and from –64.80 to –7.43 for MDF in the FMT group, compared to from –9.35 to –3.17 for MELD, from –4.45 to +1.37 for CPT, and from –58.05 to –8.33 for MDF in the SOC group (Supplementary Table S4). No information was provided on liver fibrosis.

AEs were reported in six studies, with a high degree of heterogeneity in both the quality and quantity of reported AEs. Five studies reported on the rate of ascites, with 50 cases in the SOC group compared to 24 in the FMT group at the end of follow-up. Five studies also reported on hepatic encephalopathy, with 43 cases in the SOC group versus 12 in the FMT group. Three studies examined the rate of infection, documenting 23 cases in the FMT and 41 in the SOC. Three studies reported spontaneous bacterial peritonitis, with seven cases in the SOC and six in the FMT. Finally, three studies examined excessive flatulence, identifying 29 cases in the FMT versus 3 in the SOC. For more details, including the baseline rates of these parameters; see Supplementary Table S5.

### 3.6. Risk of Bias Assessment and Level of Evidence

**Figure 4:**
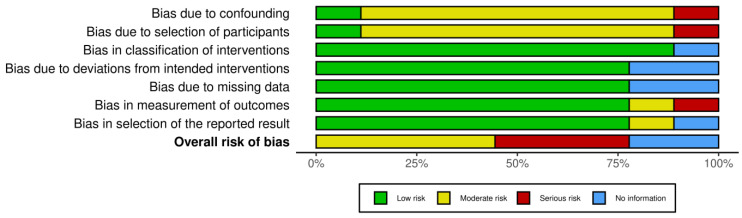
Robvis summary plot depicting the risk of bias in non-randomized included studies.

The results of ROB are summarized in Figure 4 and Supplementary Tables S6–S9. According to the ROBINS-I tool, four studies exhibited a moderate ROB, and two showed severe risk. The elevated ROB was primarily due to participant selection and confounding factors, as the studies predominantly were retrospective in design and did not include clear reporting on controlling for confounders. Of the RCTs, one study exhibited a low ROB, while another raised some concerns. The ROB for the studies by Kumar et al. [41] and Bystrianska et al. could not be adequately evaluated because they were published only as conference abstracts. The GRADEpro assessment of the level of evidence indicated very low levels for all outcomes due to high ROB in all outcomes, with the exception of overall survival, where the level of evidence was low as a result of strong association (Supplementary Table S10).

### 3.7. Publication Bias and Heterogeneity

As no analysis included more than 10 studies, small study bias could not be reliably assessed in any of the outcomes by Egger’s test. Heterogeneity was generally low with a point estimate of 0%, CI: (0 to 75%) for main outcome. Higher levels of heterogeneity were observed in the SOC group, reflecting the clinical heterogeneity of the population included.

## 4. Discussion

In this study, we review the application of FMT in ALD. To date, beyond SAH, only one study has examined the use of FMT in cirrhosis [40]. Accordingly, this article focuses on FMT for SAH. We found that FMT was associated with a twofold improvement in survival compared to SOC in SAH. Subgroup analysis by type of SOC showed that FMT was significantly superior to nutritional support and pentoxifylline treatments, but not significantly superior to glucocorticoids. Furthermore, patients with SAH exhibited a notably different microbiota composition.

Animal models have demonstrated that alterations in intestinal microbiota play a crucial role in the development and progression of AH [50]. In addition, fecal transplantation from humans with ALD, especially those with AH, into mice has been shown to increase their susceptibility to alcohol-induced liver injury [51]. Several authors have suggested that targeting the gut microbiota may be beneficial for patients suffering from AH [8,9]. Our study observed a consistent survival benefit associated with FMT, with a twofold reduction in the hazard of death. Survival benefit was observed across all included studies, with the exception of Kumar et al. [41]. In their abstract, addition of FMT to SOC was associated with higher rates of sepsis and mortality; however, the reported sample size was limited to 10 cases per arm. The greatest survival benefit was observed in patients who were either ineligible for steroids or did not respond to glucocorticoids therapy and were instead treated with pentoxifylline or received only nutritional support. A comparison of FMT to glucocorticoids therapy showed a survival trend favoring it, but this did not reach statistical nor clinical significance.

A sensitivity analysis was conducted using the Loo approach. The results revealed that only one study, [42], was particularly influential. As this was the only RCT included in the analysis, this is likely the primary reason for the significant difference observed compared to the other publications. As this study reported the smallest effect size of the included studies, it raises the question of whether the lack of randomization and blinding in other studies may have influenced the overall effect size; see those limits on the level of evidence in Supplementary Figure S2.

Although FMT is a well-tolerated intervention in most of the clinical settings [52,53] ALD and SAH represent a unique condition with an altered immune status, increased gut permeability, and susceptibility to infection; therefore, caution is warranted when considering FMT in these patients [54]. FMT is considered to be safe, with a low incidence of AEs, even in immunocompromised populations, including those with inflammatory bowel disease, C. difficile infections, and graft-versus-host disease or HIV patients [55,56,57,58,59].

Adverse events could not be synthesized because of the nature of the available data and heterogeneity in reporting methods. In the reported articles, no significant difference in serious AEs was detected between FMT and SOC. However, given the limitations of AE reporting, no definitive conclusions can be drawn. Future studies should utilize, higher-quality AE reporting. This is particularly important because commonly reported events may occur, resolve, or even precede the intervention independent of FMT, and these outcomes are also susceptible to substantial survival bias.

Overall, severity scores, such as MDF, MELD, and CPT, demonstrated comparable baseline values across the studies, suggesting a comparable population across the study arms. It has been shown that, both increasing and decreasing MELD scores over time in patients on the liver transplant waiting list reflect increased or decreased risk of death and provide valuable additional prognostic information [60]. Sharma et al. [48] and Pande et al. [42] reported a decrease in MELD at 90-day follow-up in both SOC and FMT. Notably, Pande et al. [42] observed a much smaller effect size in the reduction of risk scores, likely due to the study focus on a different patient population, as Sharma et al. [48] exclusively included patients with Acute-on-Chronic Liver Failure (see Supplementary Table S4).

### 4.1. Strengths and Limitations

This is the first meta-analysis of IPD estimates focusing on the application of FMT in the context of SAH. We synthesized all available survival data based on IPD estimates from Kaplan–Meier curves. With this approach, we achieved a relatively large sample size and a more complex analysis. The analysis was validated by running two independent models: one by pooling the re-calculated HRs and another by performing IPD analysis. Moreover, all data were cross-validated with the reported HRs in the literature when available. In addition, the protocol preregistered with Prospero was strictly followed.

In terms of limitations, studies from only three countries were included in this paper, with the vast majority of patients coming from India, which limits its applicability to the global population. Moreover, the population consists almost exclusively of male subjects.

SAH is a clinically highly diverse disease, encompassing patient groups ranging from those with relatively stable conditions to those with acute-on-chronic liver failure, as well as subgroups of patients who are responders, non-responders to treatment, or either eligible or ineligible for glucocorticoids treatment, each with a different prognosis. Although this study included a relatively large population of SAH cases, including all these subgroups, further studies with larger populations are needed, given this clinical heterogeneity, to draw reliable conclusions for those subgroups.

### 4.2. Implications for Practice and Research

Patients who are non-responders to, or ineligible for, glucocorticoids may be considered candidates for FMT; however, its clinical application should currently remain restricted given the moderate-to-high risk of bias across included studies, low certainty of evidence, and inadequate reporting of adverse events. For glucocorticoid responders, available data provide no evidence of additional benefit over conventional treatment. FMT should therefore be evaluated in further well-designed randomized controlled trials with rigorous adverse event reporting. The potential role of FMT as add-on therapy to corticosteroids warrants dedicated investigation in future trials.

The ALD spectrum, including patients with cirrhosis, steatosis, and less severe forms of AH, should also be a focus of future research. High-quality RCTs are needed to strengthen the evidence level, as only one RCT has been conducted on FMT application in SAH, making these results susceptible to confounding. Additionally, more extensive validation studies outside of South Asia are needed to expand the applicability of the findings, as prompt translation of scientific findings into practice is crucial [61,62].

## 5. Conclusions

Before FMT can be widely adopted in clinical practice, more randomized controlled trials and improved reporting of adverse events are needed. Nonetheless, it appears to be a promising therapeutic option for patients ineligible for or unresponsive to corticosteroid therapy, offering short- and mid-term survival benefits.

## Figures and Tables

**Table 1:** Basic characteristics of included studies.

Author (Year)	Study Site	Design	No of Patients (Male %)	Age (Year and SD) ‡		Liver Disease (Diagnostic Criteria)	MELD at Baseline in FMT VS SOC (Mean Difference,95 CI)	SOC (Donors)	FMT Administration Dosage	Length of FMT (Days) ATB Use	Reported Outcomes
				FMT	SOC						
Sharma et al. (2022) [48]	India	Pros	33 (100%)	39.6 (34.45–44.78) †	40.7 (37.63–43.77) †	SAH/ACLF (NIAAA)	−0.32 (−2.3; 1.7)	Nut (FM)	NSJ 30 g	Single dosage YES	Survival LFTs, AEs LFTs,
Pande et al. (2023) [42]	India	RCT	121 (97.5%)	43.15 (8.73)	40.8 (7.91)	SAH (NIAAA)	0.28 (−0.6; 1.2)	Pred (HV *)	NSD 30 g	7 Per need	Survival, RAU, LFTs, AEs LFTs, Microbiota
Philips et al. (2018) [47]	India	Retro	51 (100%)	47.6 (8,2)	47.9 (9.76)	SAH (NA)	NA	Pred, PTX, Nut (FM)	NSD 100 mL	7 Per need	Survival, LFTs, Microbiota
Philips et al. (2022) [45]	India	Retro	72 (100%)	45.2 (10.2)	47.7 (9.9)	SAH (histology)	−0.10 (2.7; 2.5)	PTX, ATB (FM)	NSD 100 mL	7 YES	Survival, RAU, AEs, Microbiota
Philips et al. (2022) [44]	India	Retro	61 (NA)	46.6 (10.1)	50.7 (11.5)	SAH (histology)	0.7 (−11.3; 12.7)	Pred (HV)	NSD 30 g	7 NA	Survival RAU AEs, Microbiota
Philips et al. (2023) [43]	India	Retro	30 (100%)	53.3 (4.1)	51.8 (6.4)	SAH (NIAAA)	NA	Pred (HV)	NSD 100 mL	7 Per need	RAU
Philips et. al (2017) [46]	India	Retro	36 (NA)	NA	NA	SAH (NIAAA)	4.0 (−0.5; 8.5)	NA (FM)	NSD 30 g	7 NA	Survival, LFTs, Microbiota
Kumar et al. (2022) [41] **‡**	India	RCT	20 (NA)	NA	NA	SAH (NA)	NA	NA (NA)	NA NA	NA NA	Survival
Bystrianska et al. (2022) [47]	Slovakia	Pros	50 (NA)	NA	NA	SAH (NA)	NA	Nut (HV)	NA 100 mL	6 NA	Survival
Bajaj et al. (2020) [40]	USA	RCT	20 (100%)	64.5 (5.1)	62.9 (9.8)	Cirrhosis (**)	−1.2 (−4.1; 1.7)	Placebo (HV)	Enema 27 g/100 mL	Single dosage NA	RAU

† parameters represented as mean with upper and lower confidence intervals. ‡ study included only in systematic review. * healthy volunteers living in the same household, ** prior diagnosis of cirrhosis with an AUDIT score equal to or more than 8. ACLF: acute-on-chronic liver failure, AEs: adverse events, ATB: antibiotics, CI: confidence interval, FMT: fecal microbiota transplantation, FM: family members, HV: healthy volunteers, LFTs: Liver Function Tests, MELD: Model for End-Stage Liver Disease, SD: standard deviation, NIAAA: National Institute on Alcohol Abuse and Alcoholism, NSD: nasoduodenal tube, NSJ: nasojejunal tube, Nut: nutritional support, Pred: Prednisone, Pro: prospective study design, PTX: Pentoxifylline, RAU: recurrence of alcohol use, RCT: randomized controlled trial, Retro: retrospective study design, SAH: severe alcoholic hepatitis, SOC: standard of care.

## Data Availability

All data used in this systematic review and meta-analysis were extracted from the published full-text articles and Supplementary Materials of the included studies, as listed in the reference list.

## Supplementary Materials

The following supporting information can be downloaded at: https://www.mdpi.com/article/10.3390/jcm15135131/s1, Supplementary Figures: Supplementary Figure S1. Funnel plot illustrating publication bias among the included studies utilizing the Corticosteroids arm in Philips et al. (2018) [47]. Supplementary Figure S2. Leave-one-out analysis and influential of the included studies utilizing the Corticosteroids arm in Philips et al. (2018) [47]. *HR: hazard ratio*. Supplementary Figure S3. Kaplan–Meier curve depicting individual patient data and pooled survival data for FMT vs. SOC utilizing the NUT arm in Philips et al. (2018) [47]. *HR: hazard ratio; FMT: Faecal Microbiota Transplantation; SOC: Standard of Care; NUT: nutritional*. Supplementary Figure S4. Forest plot showing the HR for FMT vs. SOC based on individual patient data utilizing the nutritional arm in Philips et al. (2018) [47]. *HR: hazard ratio; FMT: Faecal Microbiota Transplantation; SOC: Standard of Care*. Supplementary Figure S5. Forest plot showing the pooled HR for FMT vs. SOC based on the re-calculated HR utilizing the nutritional arm in Philips et al. (2018) [47]. *HR: hazard ratio; FMT: Faecal Microbiota Transplantation; SOC: Standard of Care*. Supplementary Figure S6. Leave-one-out analysis and influential of the included studies utilizing the nutritional arm in Philips et al. (2018) [47]. *HR: hazard ratio*. Supplementary Figure S7. Funnel plot illustrating publication bias among the included studies utilizing the utilizing the nutritional arm in Philips et al. (2018) [47]. Supplementary Figure S8. Kaplan–Meier curve depicting individual patient data and pooled survival data for FMT vs. SOC, utilizing the PTX arm in Philips et al. (2018) [47]. *PTX: pentoxifylline; FMT.* Supplementary Figure S9. Forest plot showing the HR for FMT vs. SOC based on individual patient data from the pentoxifylline arm in Philips et al. (2018) [47]. *HR: hazard ratio; FMT: Faecal Microbiota Transplantation; SOC: Standard of Care*. Supplementary Figure S10. Forest plot showing the pooled HR for FMT vs. SOC based on the re-calculated HR utilizing the pentoxifylline arm in Philips et al. (2018) [47]. *HR: hazard ratio; FMT: Faecal Microbiota Transplantation; SOC: Standard of Care*. Supplementary Figure S11. Leave-one-out analysis and influential of the included studies utilizing the pentoxifylline arm in Philips et al. (2018) [47]. Supplementary Figure S12. Funnel plot illustrating publication bias among the included studies utilizing the pentoxifylline arm in Philips et al. (2018) [47]. Supplementary Figure S13. Forest plot depicting the 28-day survival probability for faecal microbiota transplantation vs. standard of care. Supplementary Figure S14. Leave-one-out analysis of the 28-day survival probability for faecal microbiota transplantation vs. standard of care. Supplementary Figure S15. Forest plot depicting the 90-day survival probability for faecal microbiota transplantation vs. standard of care. Supplementary Figure S16. Forest plot depicting the 180-day survival probability for faecal microbiota transplantation vs. standard of care. Supplementary Figure S17. Forest plot depicting the 28-day survival probability for faecal microbiota transplantation vs. standard of care in a model utilizing the nutritional arm in Philips et al. (2018) [47]. Supplementary Figure S18. Leave-one-out analysis of the 28-day survival probability for faecal microbiota transplantation vs. standard of care in a model utilizing the nutritional arm in Philips et al. (2018) [47]. Supplementary Figure S19. Forest plot depicting the 90-day survival probability for faecal microbiota transplantation vs. standard of care in model utilizing the nutritional arm in Philips et al. (2018) [47]. Supplementary Figure S20. Forest plot depicting the 180-day survival probability for faecal microbiota transplantation vs. standard of care in a model utilizing the nutritional arm in Philips et al. (2018) [47]. Supplementary Figure S21. Forest plot depicting the 28-day survival probability for faecal microbiota transplantation vs. standard of care in a model utilizing the pentoxifylline arm in Philips et al. (2018) [47]. Supplementary Figure S22. Leave-one-out analysis of the 28-day survival probability for faecal microbiota transplantation vs. standard of care in a model utilizing the pentoxifylline arm in Philips et al. (2018) [47]. Supplementary Figure S23. Forest plot depicting the 90-day survival probability for faecal microbiota transplantation vs. standard of care in a model utilizing the pentoxifylline arm in Philips et al. (2018) [47]. Supplementary Figure S24. Forest plot depicting the 180-day survival probability for faecal microbiota transplantation vs. standard of care in a model utilizing the pentoxifylline arm in Philips et al. (2018) [47]. Supplementary Figure S25. Forest plot depicting the MD of MELD score at baseline between faecal microbiota transplantation vs. standard of care arms of included studies. *MD: mean difference, MELD: model for End-Stage Liver Disease*. Supplementary Figure S26. Forest plot depicting the MD of CPT score at baseline between FMT and SOC arm in included studies. *CPT: Child–Pugh–Turcotte, MD: mean difference*. Supplementary Figure S27. Forest plot depicting the MD of MDF score at baseline between FMT and SOC arm in included studies. *MD: mean difference; MDF: Maddrey’s Discriminant Function.* Table & Legends: Supplementary Table S1. PRISMA checklist. *From:* Page MJ, McKenzie JE, Bossuyt PM, Boutron I, Hoffmann TC, Mulrow CD, et al. The PRISMA 2020 statement: an updated guideline for reporting systematic reviews. BMJ 2021;372:n71. doi: 10.1136/bmj.n71. This work is licensed under CC BY 4.0. To view a copy of this license, visit https://creativecommons.org/licenses/by/4.0/. Supplementary Table S2: Pairwise Comparisons of HRs for All Treatment Arms with Tukey Multiplicity Corrected p-values and CI (Calculated on Log-Scale) 95CI: 95% Confidence Interval, CS: Glucocorticoids, FMT: Fecal Microbiota Transplantation, HR: Hazard Ratio, NUT: Nutritional Support Only, PTX: Pentoxifylline. Supplementary Table S3. Overview of the microbiota data reported in the included studies. FMT: Faecal Microbiota Transplantation, SOC: Standard of Care. Supplementary Table S4. Severity Score Comparisons at Baseline during Follow-ups and difference between baseline and end of follow up. *CPT: Child–Pug–Turcotte h, FMT: Fecal Microbiota Transplantation, MELD: Model for End-Stage Liver Disease, MDF: Maddrey Discriminant Function, SD: Standard Deviation, SE: Standard Error, SOC: Standard of Care, *mean and SD*. Supplementary Table S5. The adverse event rate at base line and follow up. FMT: Faecal Microbiota Transplantation, SOC: Standard of Care. Supplementary Table S6. Robvis traffic light plot depicting the risk of bias in non-randomized studies. Supplementary Table S7. Robvis summary plot depicting the risk of bias in non-randomized included studies. Supplementary Table S8. Robvis traffic light plot depicting RoB 2 in randomized controlled trials of included studies for survival outcomes. Supplementary Table S9. Robvis traffic light plot depicting RoB 2 in RCTs of included studies for abstinence after treatment. Supplementary Table S10. Summary of the quality of evidence by GRADEpro for survival outcomes and recurrence of alcoholism after treatment. CI: confidence interval; HR: hazard ratio; OR: odds ratio.

## Abbreviations

The following abbreviations are used in this manuscript:

AEs	Adverse events
AH	Alcoholic hepatitis
ALD	Alcohol-related liver disease
CPT	Child-Pugh Score
CI	Confidence interval
DSC	Disease severity scores
FMT	Fecal microbiota transplantation
HR	Hazard ratio
IPD	Individual patient data
Loo	Leave-one-out
MDF	Maddrey’s Discriminant Function
MELD	Model for End-Stage Liver Disease
RCT	Randomized controlled trial
ROB	Risk of bias
SAH	Severe alcoholic hepatitis
SOC	Standard of care
