# Supplementary material for: Fecal Microbiota Transplantation Is Associated with Better Survival Compared to Standard of Care in Severe Alcoholic Hepatitis Not Responding to Corticosteroids: A Systematic Review and Meta-Analysis
Source: J Clin Med. 2026 Jul 1;15(13):5131. doi: 10.3390/jcm15135131 (PMC13363518; doi:10.3390/jcm15135131)
Supplement: Supplementary file 1 [file jcm-15-05131-s001.zip › jcm-4312916-supplementary.pdf]

Supplementary material

Fecal Microbiota Transplantation Is Associated with Better Survival Compared to Standard of Care in Severe Alcoholic Hepatitis Not Responding to Corticosteroids: A Systematic Review and Meta-Analysis

Authors

Jakub Hoferica[1,2], Bettina Csilla Budai[1,3], Eszter Ágnes Szalai[1,4], Ádám Zolcsák[1,5], Marie Anne Engh[1], Katalin Lenti [1,6], Péter Hegyi[1,3,7,8], Jun Yu[9],Péter Jenő Hegyi[1,3]*, Peter Banovcin[1,2]*

Affiliations:

1 Centre for Translational Medicine, Semmelweis University, Budapest, Hungary

2 Jessenius Faculty of Medicine in Martin, Comenius University, Martin, Slovakia

3 Institute of Pancreatic Diseases, Semmelweis University, Budapest, Hungary

4 Department of Restorative Dentistry and Endodontics, Semmelweis University, Budapest, Hungary

5 Department of Biophysics and Radiation Biology, Semmelweis University, Budapest, Hungary

6 Department of Morphology and Physiology, Faculty of Health Sciences, Semmelweis University, Budapest, Hungary

7 Institute for Translational Medicine, Medical School, University of Pécs, Pécs, Hungary.

8 Translational Pancreatology Research Group, Interdisciplinary Centre of Excellence for Research Development and Innovation University of Szeged, Szeged, Hungary

9 Chinese University of Hong Kong, Hong Kong, SAR

Corresponding author

doc. MUDr. Peter Bánovčin, Ph.D., MBA

Postal address: Kollárova 2, 036 59 Martin, Slovakia

Tel.: +421 43 4203 266

E-mail address: peter.banovcin2@uniba.sk

Addition synthesis methods

Search strategy

Supplementary Figure S1. Funnel plot illustrating publication bias among the included studies utilizing the Corticosteroids arm in Philips et al. (2018).

Supplementary Figure S2. Leave-one-out analysis and influential of the included studies utilizing the Corticosteroids arm in Philips et al. (2018).

Supplementary Figure S3. Kaplan–Meier curve depicting individual patient data and pooled survival data for FMT vs. SOC utilizing the NUT arm in Philips et al. (2018).

Supplementary Figure S4. Forest plot showing the HR for FMT vs. SOC based on individual patient data utilizing the nutritional arm in Philips et al. (2018).

Supplementary Figure S5. Forest plot showing the pooled HR for FMT vs. SOC based on the re-calculated HR utilizing the nutritional arm in Philips et al. (2018).

Supplementary Figure S6. Leave-one-out analysis and influential of the included studies utilizing the nutritional arm in Philips et al. (2018).

Supplementary Figure S7. Funnel plot illustrating publication bias among the included studies utilizing the utilizing the nutritional arm in Philips et al. (2018).

Supplementary Figure S8. Kaplan–Meier curve depicting individual patient data and pooled survival data for FMT vs. SOC, utilizing the PTX arm in Philips et al. (2018).

Supplementary Figure S9. Forest plot showing the HR for FMT vs. SOC based on individual patient data from the pentoxifylline arm in Philips et al. (2018).

Supplementary Figure S10. Forest plot showing the pooled HR for FMT vs. SOC based on the re-calculated HR utilizing the pentoxifylline arm in Philips et al. (2018).

Supplementary Figure S11. Leave-one-out analysis and influential of the included studies utilizing the pentoxifylline arm in Philips et al. (2018).

Supplementary Figure S12. Funnel plot illustrating publication bias among the included studies utilizing the pentoxifylline arm in Philips et al. (2018).

Supplementary Figure S13. Forest plot depicting the 28-day survival probability for faecal microbiota transplantation vs standard of care.

Supplementary Figure S14. Leave-one-out analysis of the 28-day survival probability for faecal microbiota transplantation vs standard of care.

Supplementary Figure S15. Forest plot depicting the 90-day survival probability for faecal microbiota transplantation vs standard of care.

Supplementary Figure S16. Forest plot depicting the 180-day survival probability for faecal microbiota transplantation vs standard of care.

Supplementary Figure S17. Forest plot depicting the 28-day survival probability for faecal microbiota transplantation vs standard of care in a model utilizing the nutritional arm in Philips et al. (2018).

Supplementary Figure S18. Leave-one-out analysis of the 28-day survival probability for faecal microbiota transplantation vs standard of care in a model utilizing the nutritional arm in Philips et al. (2018).

Supplementary Figure S19. Forest plot depicting the 90-day survival probability for faecal microbiota transplantation vs standard of care in model utilizing the nutritional arm in Philips et al. (2018).

Supplementary Figure S20. Forest plot depicting the 180-day survival probability for faecal microbiota transplantation vs standard of care in a model utilizing the nutritional arm in Philips et al. (2018).

Supplementary Figure S21. Forest plot depicting the 28-day survival probability for faecal microbiota transplantation vs standard of care in a model utilizing the pentoxifylline arm in Philips et al. (2018).

Supplementary Figure S22. Leave-one-out analysis of the 28-day survival probability for faecal microbiota transplantation vs standard of care in a model utilizing the pentoxifylline arm in Philips et al. (2018).

Supplementary Figure S23. Forest plot depicting the 90-day survival probability for faecal microbiota transplantation vs standard of care in a model utilizing the pentoxifylline arm in Philips et al. (2018).

Supplementary Figure S24. Forest plot depicting the 180-day survival probability for faecal microbiota transplantation vs standard of care in a model utilizing the pentoxifylline arm in Philips et al. (2018).

Supplementary Figure S25. Forest plot depicting the MD of MELD score at baseline between faecal microbiota transplantation vs standard of care arms of included studies.

Supplementary Figure S26. Forest plot depicting the MD of CPT score at baseline between FMT and SOC arm in included studies.

Supplementary Figure S27. Forest plot depicting the MD of MDF score at baseline between FMT and SOC arm in included studies.

Table & Legends

Supplementary Table S1. PRISMA checklist

Supplementary Table S2. Pairwise Comparisons of HRs for All Treatment Arms with Tukey Multiplicity Corrected p-values and CI (Calculated on Log-Scale)

Supplementary Table S3. Overview of the microbiota data reported in the included studies

Supplementary Table S4. Severity Score Comparisons at Baseline during Follow-ups and difference between baseline and end of follow up

Supplementary Table S5. The adverse event rate at base line and follow up

Supplementary Table S6. Robvis traffic light plot depicting the risk of bias in non-randomized studies

Supplementary Table S7. Robvis summary plot depicting the risk of bias in non-randomized included studies

Supplementary Table S8. Robvis traffic light plot depicting RoB 2 in randomized controlled trials of included studies for survival outcomes

Supplementary Table S9. Robvis traffic light plot depicting RoB 2 in RCTs of included studies for abstinence after treatment

Supplementary Table S10. Summary of the quality of evidence by GRADEpro for survival outcomes and recurrence of alcoholism after treatment

Synthesis methods

As we assumed considerable heterogeneity among the underlying population of the reported study results due to the nature of the examined question, random effects models were used in a frequentist framework. The minimum number of studies required for analysis was 3. In the case of any overlapping population, the study with large samples size or longer follow-up was used.

For time-to-event data, we used hazard ratios (HRs) with a 95% confidence interval (CI) as the main measure of effect between the two groups: FMT and SOC. We calculated HR as FMT vs. SOC groups. To calculate the pooled HR, we used two methods: (1) a classical random-effects meta-analysis method (hereafter referred to as the 'classical' method) based on calculated study HRs, and (2) an individual patient data (IPD)-based random-effects Cox hazard model with Gaussian random effects (hereafter referred to as 'IPD-based'). Additionally, as a secondary measure, we estimated survival probabilities at specific time points and pooled them with a 3-level model ("multilevel") to indirectly compare survival in the groups. An estimation for distribution-free pooled survival curves was also performed using the method implemented by Pandey [1] ("curve estimate"). An additional analysis was performed using the IPD-based model to compare the HR in the experimental (FMT) and separately in the three different control groups (nutritional support (Nut) and pentoxifylline (PTX), CS).

When articles did not report HR and used varying outcome measures for time-to-event data, but provided Kaplan–Meier curves, we derived IPD estimates from these plots. We used the free WebPlotDigitizer tool to extract values from the plots[2]. We compared the extracted and calculated data with the published data where possible.

Results were considered statistically significant if the pooled CI does not contain the null value. We summarized the findings in tables, in forest plots, Kaplan-Meier plots (using Kaplan-Meier estimates), and estimated survival curves. Where appropriate, between-study heterogeneity was described by the between-study variance ($\tau 2$) and also I^2 statistics based on the classical method, and the 3-level method. For IPD-based results, we reported variance of the random-effects. Small study publication bias in the classical and multilevel methods was assessed by visual inspection of Funnel-plots and calculating Egger's test p-value [3] using a 10% significance level. However, we assumed possible small study bias based on the p-value if the study number was at least 10. Potential outlier publications were explored using different influence measures and plots following the recommendation of Harrer et. al. [4]

All statistical analyses were calculated by R software [5] using the meta[6,7] (v7.0.0) package for basic meta-analysis calculations and plots, and the dmetar [8](v0.1.0) package for additional influential analysis calculations and plots for the classical method. The package metafor [9] (v4.4.0) was used for the multilevel model. The packages survival [10] [11] (v3.7.0), survminer [12](v0.4.9) and coxme [13] (v2.2.20) were used for IPD-based calculations. MetaSurvival [1] (v0.1.0) package was used with the ecurve estimation method.

In case of the classical method, for pooling the effect size, inverse variance method was used. We used a Hartung-Knapp adjustment[14] [15]for CIs. This adjustment was applied only if it is more conservative then the classical one (as recommended in Jackson et al. [16] as hybrid method 2). To estimate the heterogeneity variance measure ($\tau2$), a restricted maximum likelihood method was used with the Q profile method for confidence interval (recommended in Harrer et al. and Veroniki et al.[4,17]). For assessing the small study publication bias, funnel-plots was used with visual inspection. Additionally, we performed Egger's[3] test, although it has limited diagnostic assessment below ~10 study. Between-study heterogeneity was additionally described by the Higgins&Thompson's I2 Statistics[18]. Potential outlier publications was explored using different influence measures and plots following the recommandation of Harrer et. al[4].

For IPD based analysis, we used a mixed effect Cox model with a single random intercept (also called frailty) model. Gaussian random effects distribution was assumed. The assumption of proportional hazard and random effects distribution, martingale residuals was examined on plots and found acceptable. As an additional sensitivity assessment, we performed a leave-one-out study analysis when comparing SOC and FMT groups.

We assumed the secondary outcomes (survival probabilities - proportions - in SOC and FMT groups) are more similar to each other for different samples within the same study than for samples from different studies. Consequently, we used an additional (independent) random effect in our analysis using a multilevel meta-analysis for pooling survival probabilities at a given time point among groups. The survival probabilities with its 95% CI were estimated pointwisely using Kaplan-Meier estimates[19] implemented in the survival R package. In the multilevel analyses we used the logit transformation of the calculated proportions. In case when the calculated proportion was 1 (or 0) we did a continuity correction with adding 0.5 to 0 event and the corresponding total sample size value than calculating the proportion with its exact binomial 95% CI. We used inverse variance weighting with restricted maximum likelihood method for pooling the proportions. For confidence interval calculation and prediction intervals we used a t-distribution method. For test of subgroup differences, a "Cochrane Q" test (an omnibus test) was used between subgroups[4]. The null hypothesis was rejected on a 5% significance level. In case of the 3 level model we reported the I2 statistics for each level with its 95% CI as given in[20]. We refer as "total I2" for the sum of the two level as the total heterogeneity over sampling variance. In the case of multilevel results, we calculated dfbetas (the pooled effect size without the given study expressed in logit scale), Cook's distances (shows how much the estimated effect size changes leaving-out the given study but taking into consideration of how much differ the leaved-out study effect size from the pooled effect size. - typical threshold for potential influential value is 2) and hat values (the value of the hat matrix without the given study.) leaving out 1 study at a time.

Survival curve estimate method was based on the article by Combescure[21] and implemented in the metaSurvival package. WE used the Greenwoods formula to estimate survival probabilities. As the sample size was 20-100/groups in the studies, to avoid the overfitting, we used only 5 time points to estimate the pooled curve.

Search strategy

The following search key was used in all three databases:

(((fecal OR faecal OR feces OR faeces OR stool) AND (microbiota OR microbiome OR bacteria OR microflora) AND (transplantation OR transplant OR transfer)) OR fmt OR (fecal microbiota transplantation)) AND (liver OR hepa* OR sah OR ald OR arld OR stea* OR alco* OR cirr* OR cirrhosis)

Supplementary Figure & Legends:

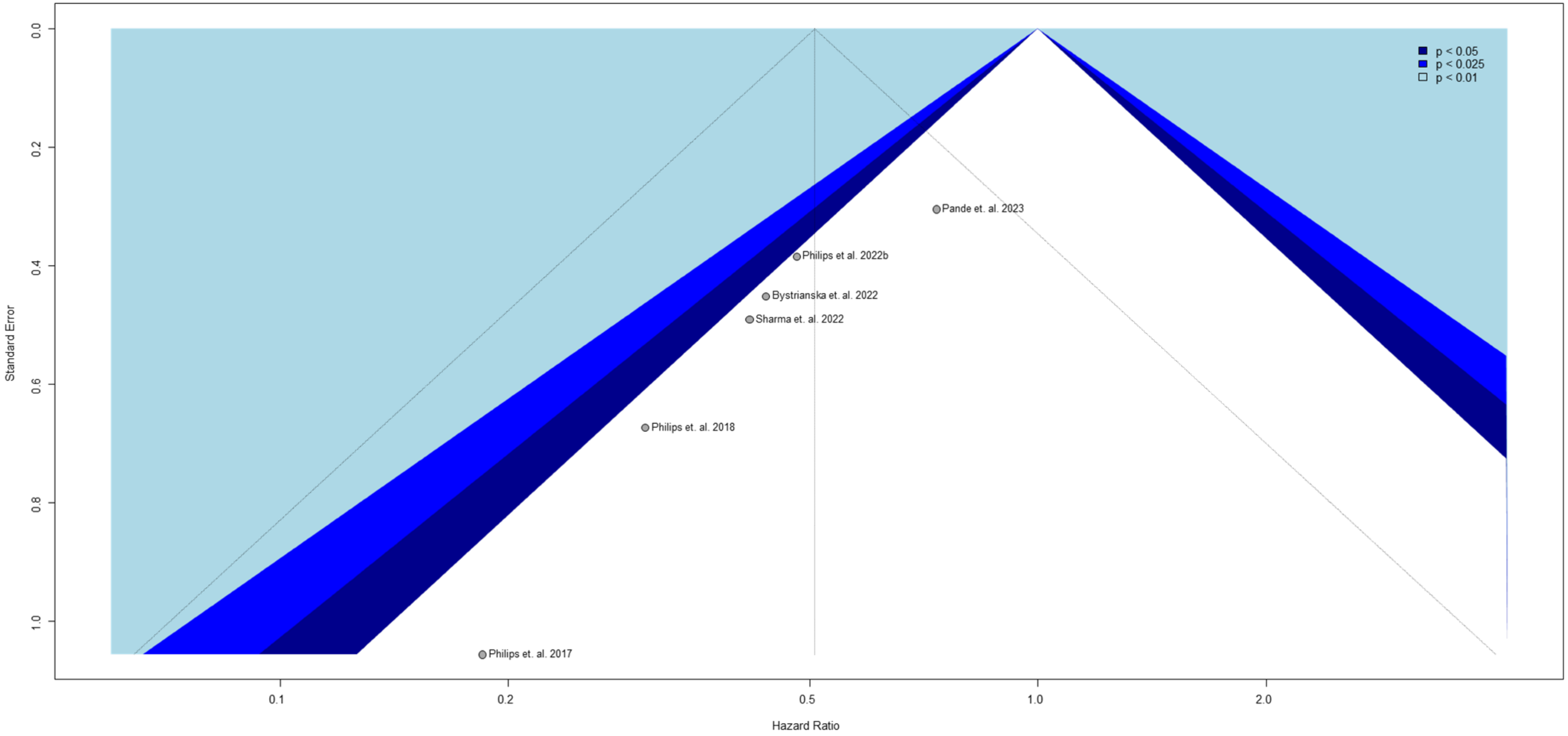

Supplementary Figure S1. Funnel plot illustrating publication bias among the included studies utilizing the Corticosteroids arm in Philips et al. (2018).

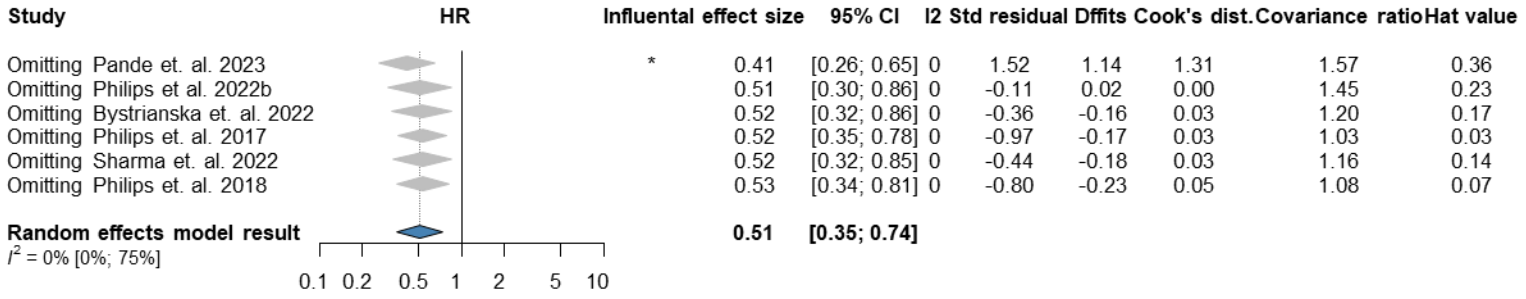

Supplementary Figure S2. Leave-one-out analysis and influential of the included studies utilizing the Corticosteroids arm in Philips et al. (2018).

HR: hazard ratio

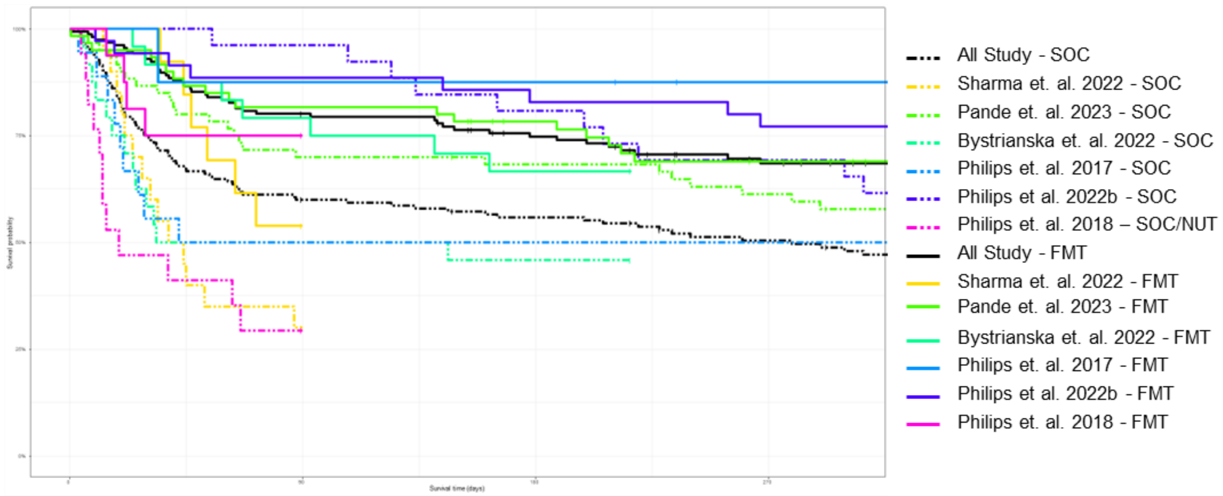

Supplementary Figure S3. Kaplan–Meier curve depicting individual patient data and pooled survival data for FMT vs. SOC utilizing the NUT arm in Philips et al. (2018).

HR: hazard ratio; FMT: Faecal Microbiota Transplantation; SOC: Standard of Care; NUT: nutritional

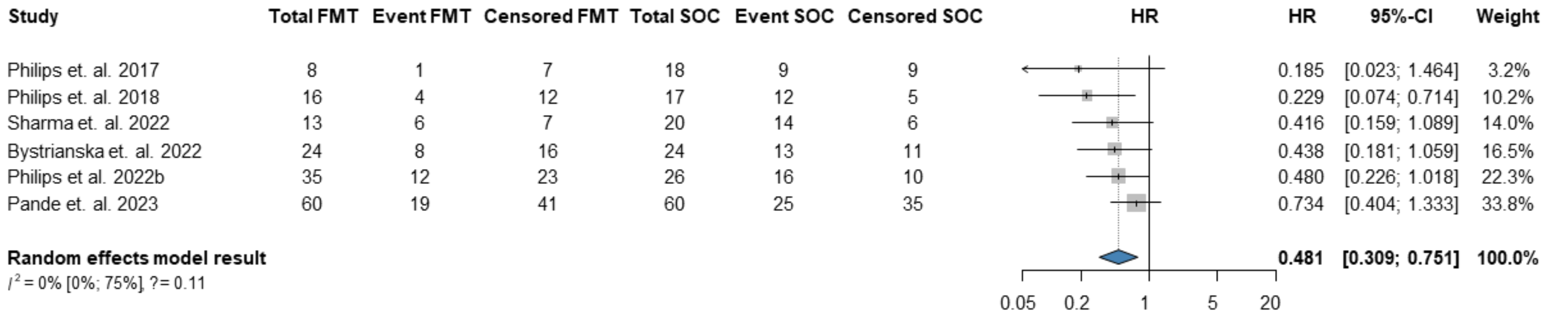

Supplementary Figure S4. Forest plot showing the HR for FMT vs. SOC based on individual patient data utilizing the nutritional arm in Philips et al. (2018).

HR: hazard ratio; FMT: Faecal Microbiota Transplantation; SOC: Standard of Care

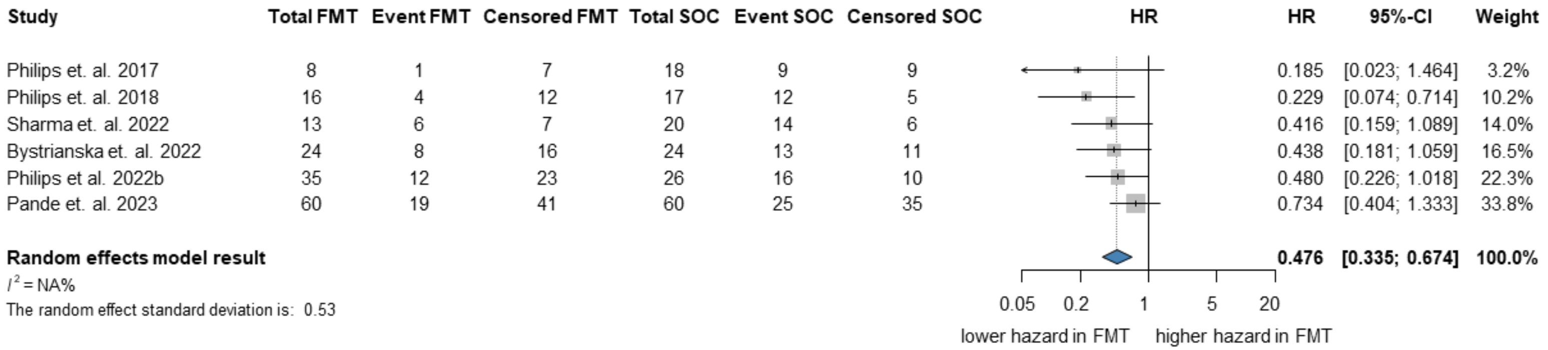

Supplementary Figure S5. Forest plot showing the pooled HR for FMT vs. SOC based on the re-calculated HR utilizing the nutritional arm in Philips et al. (2018).

HR: hazard ratio; FMT: Faecal Microbiota Transplantation; SOC: Standard of Care

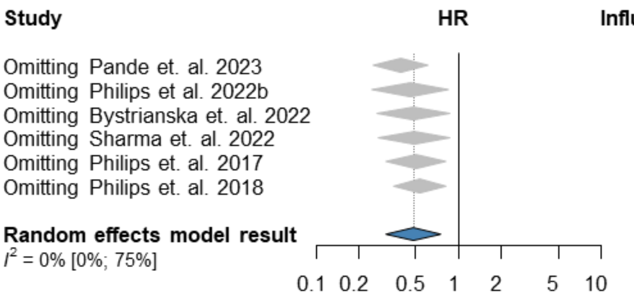

Study	Influental	effect size	95% CI	I2	Std residual	Dffits	Cook's dist.	Covariance ratio	Hat value
Omitting Pande et. al. 2023	*	0.39	[0.25; 0.61]	0.00	1.67	1.19	1.25	1.43	0.34
Omitting Philips et al. 2022b		0.46	[0.25; 0.85]	0.11	0.09	0.24	0.08	1.71	0.22
Omitting Bystrianska et. al. 2022		0.47	[0.26; 0.86]	0.10	-0.15	0.07	0.01	1.46	0.16
Omitting Sharma et. al. 2022		0.48	[0.27; 0.86]	0.09	-0.26	-0.01	0.00	1.35	0.14
Omitting Philips et. al. 2017		0.50	[0.31; 0.81]	0.00	-0.93	-0.20	0.04	0.98	0.03
Omitting Philips et. al. 2018		0.53	[0.34; 0.81]	0.00	-1.37	-0.51	0.25	1.02	0.10
**Random effects model result**		**0.48**	**[0.31; 0.75]**						

I^2 = 0% [0%; 75%]

Supplementary Figure S6. Leave-one-out analysis and influential of the included studies utilizing the nutritional arm in Philips et al. (2018).

HR: hazard ratio

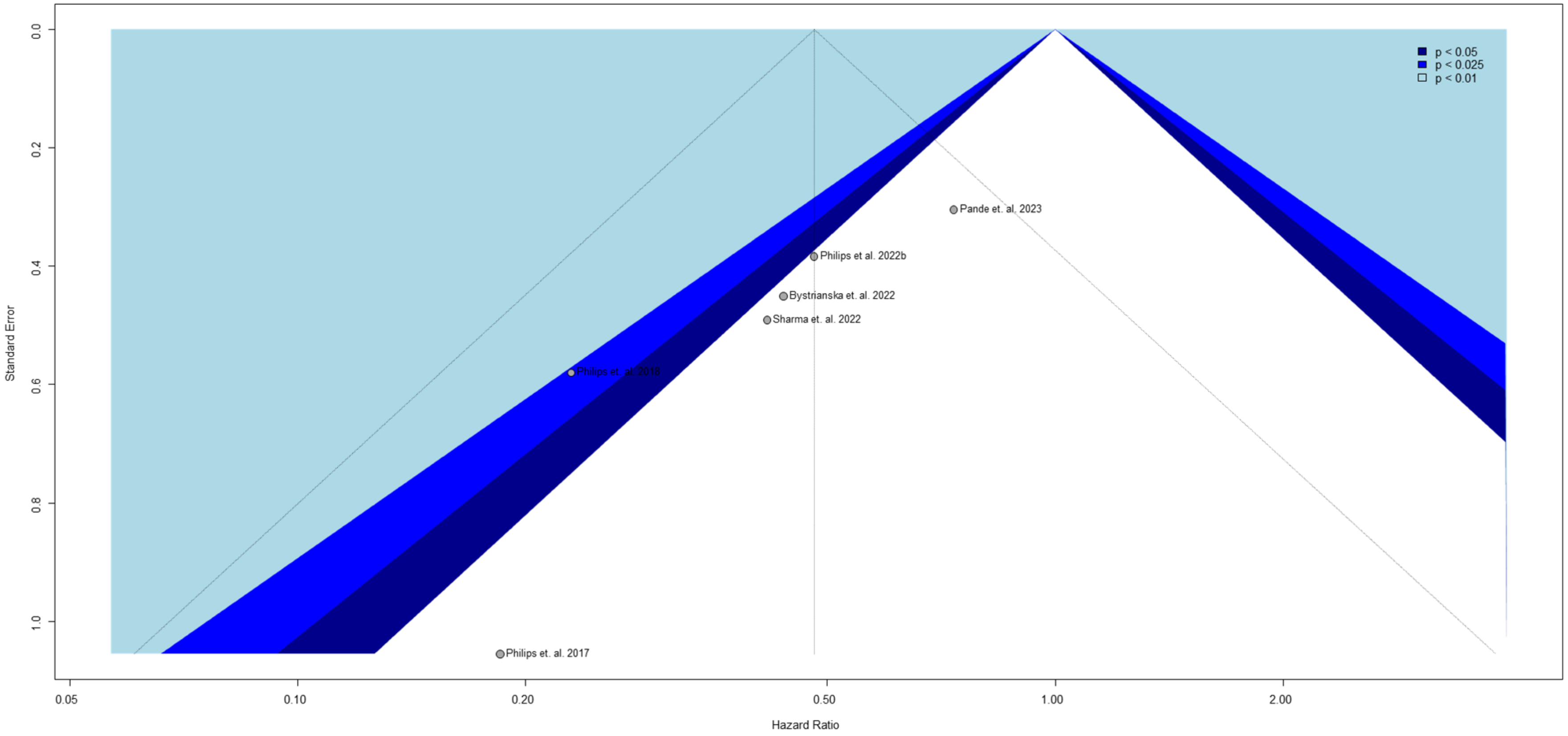

Supplementary Figure S7. Funnel plot illustrating publication bias among the included studies utilizing the utilizing the nutritional arm in Philips et al. (2018).

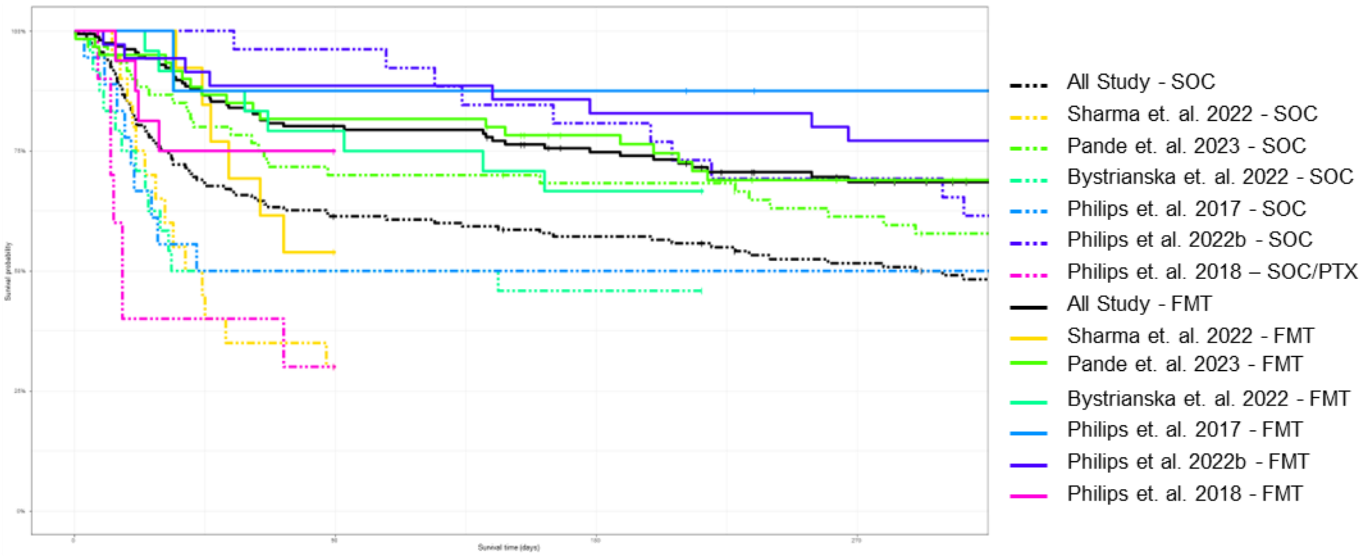

Supplementary Figure S8. Kaplan–Meier curve depicting individual patient data and pooled survival data for FMT vs. SOC, utilizing the PTX arm in Philips et al. (2018).

PTX: pentoxifylline; FMT

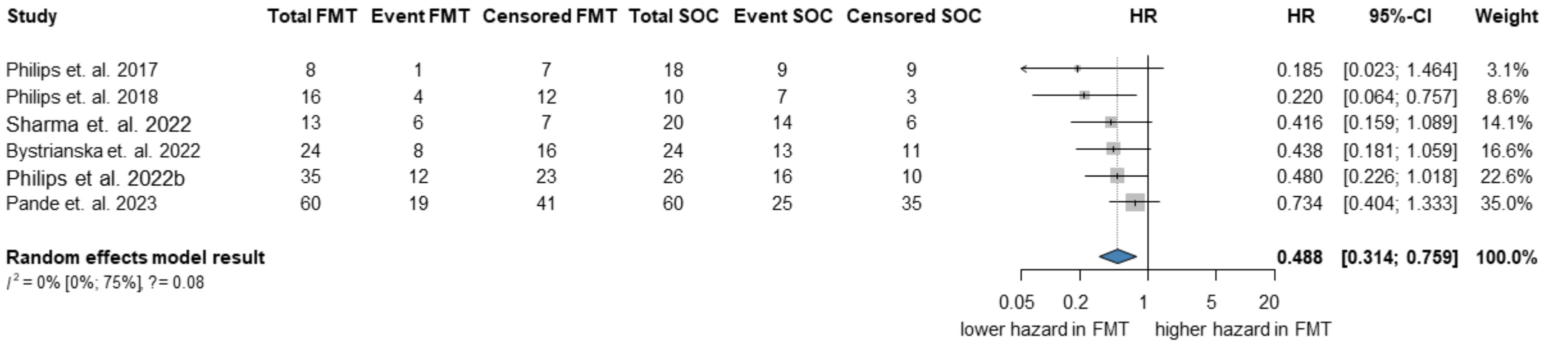

Supplementary Figure S9. Forest plot showing the HR for FMT vs. SOC based on individual patient data from the pentoxifylline arm in Philips et al. (2018).

HR: hazard ratio; FMT: Faecal Microbiota Transplantation; SOC: Standard of Care

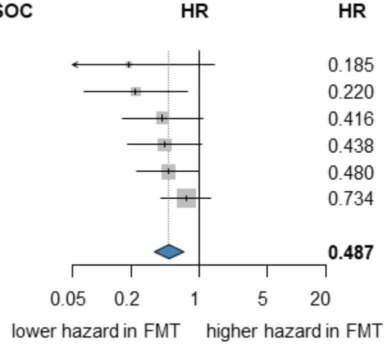

Study	Total FMT	Event FMT	Censored FMT	Total SOC	Event SOC	Censored SOC	HR	95%-CI	Weight
Philips et. al. 2017	8	1	7	18	9	9	0.185	[0.023; 1.464]	3.1%
Philips et. al. 2018	16	4	12	10	7	3	0.220	[0.064; 0.757]	8.6%
Sharma et. al. 2022	13	6	7	20	14	6	0.416	[0.159; 1.089]	14.1%
Bystrianska et. al. 2022	24	8	16	24	13	11	0.438	[0.181; 1.059]	16.6%
Philips et al. 2022b	35	12	23	26	16	10	0.480	[0.226; 1.018]	22.6%
Pande et. al. 2023	60	19	41	60	25	35	0.734	[0.404; 1.333]	35.0%
**Random effects model result**							**0.487**	**[0.342; 0.694]**	**100.0%**

I^2 = NA%

The random effect standard deviation is: 0.5

Supplementary Figure S10. Forest plot showing the pooled HR for FMT vs. SOC based on the re-calculated HR utilizing the pentoxifylline arm in Philips et al. (2018).

HR: hazard ratio; FMT: Faecal Microbiota Transplantation; SOC: Standard of Care

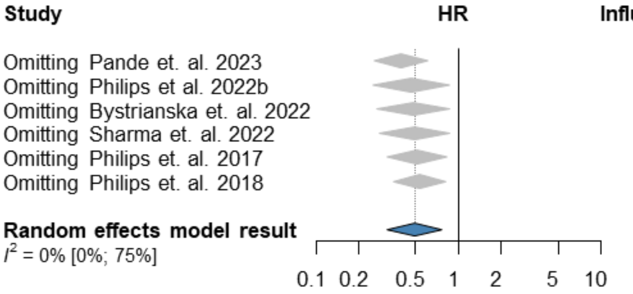

Study	Influental	effect size	95% CI	I2	Std residual	Dffits	Cook's dist.	Covariance ratio	Hat value
Omitting Pande et. al. 2023	*	0.39	[0.25; 0.61]	0.00	1.64	1.20	1.35	1.49	0.35
Omitting Philips et al. 2022b		0.46	[0.25; 0.86]	0.09	0.07	0.24	0.08	1.74	0.23
Omitting Bystrianska et. al. 2022		0.48	[0.27; 0.88]	0.07	-0.18	0.06	0.00	1.46	0.17
Omitting Sharma et. al. 2022		0.49	[0.27; 0.87]	0.06	-0.29	-0.02	0.00	1.33	0.14
Omitting Philips et. al. 2017		0.51	[0.31; 0.82]	0.00	-0.94	-0.20	0.04	0.98	0.03
Omitting Philips et. al. 2018		0.53	[0.34; 0.81]	0.00	-1.33	-0.44	0.19	1.04	0.09
**Random effects model result**		**0.49**	**[0.31; 0.76]**						

I^2 = 0% [0%; 75%]

Supplementary Figure S11. Leave-one-out analysis and influential of the included studies utilizing the pentoxifylline arm in Philips et al. (2018).

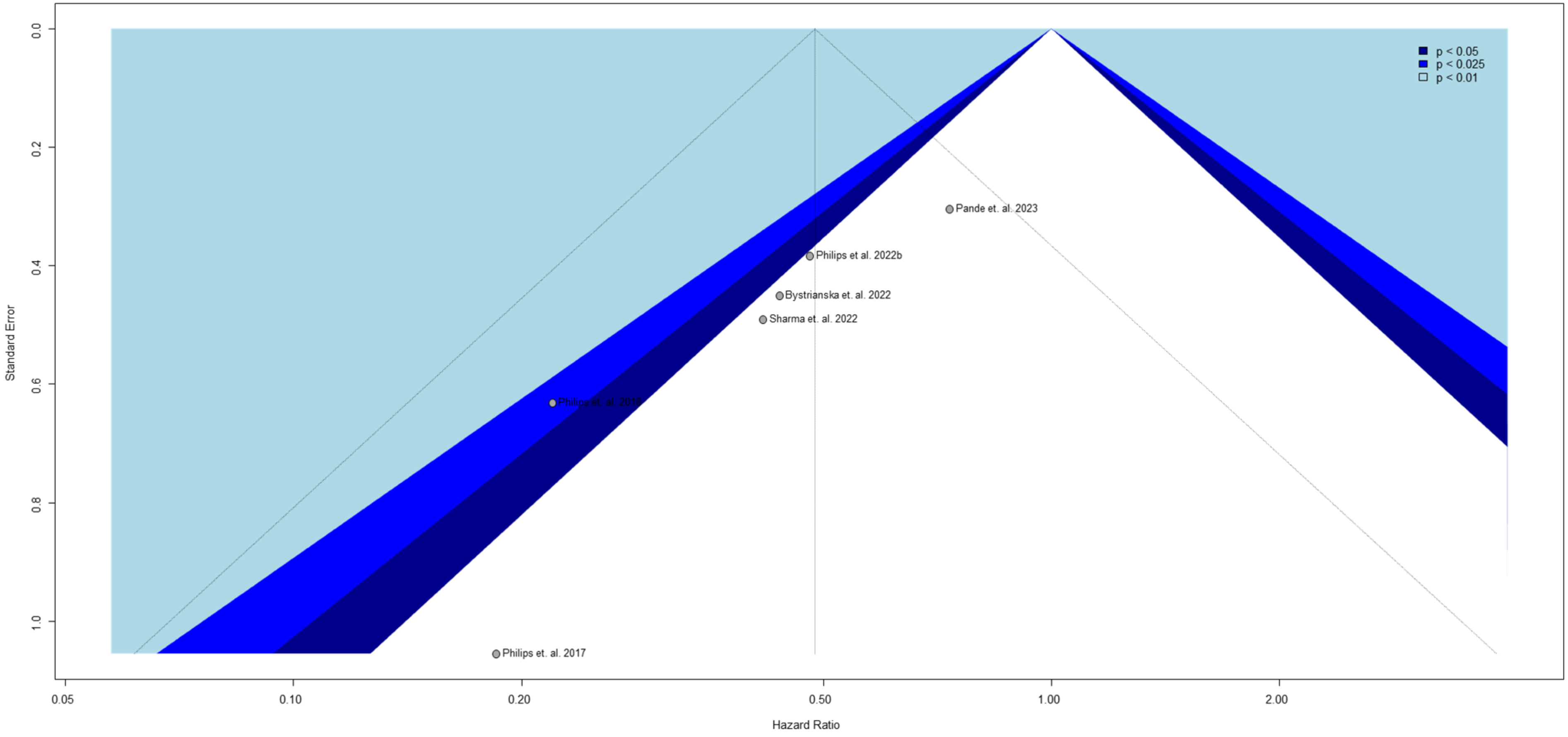

Supplementary Figure S12. Funnel plot illustrating publication bias among the included studies utilizing the pentoxifylline arm in Philips et al. (2018).

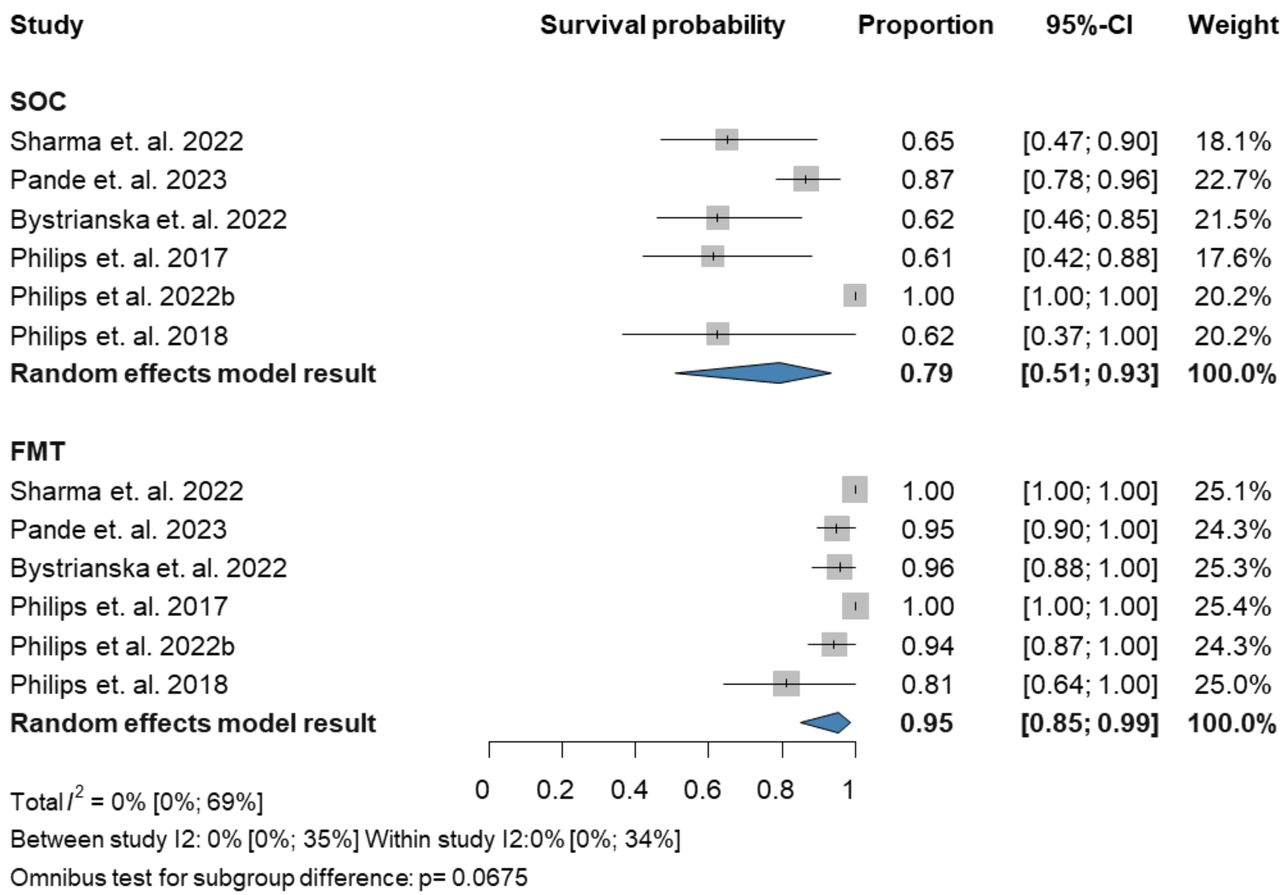

Supplementary Figure S13. Forest plot depicting the 28-day survival probability for faecal microbiota transplantation vs standard of care.

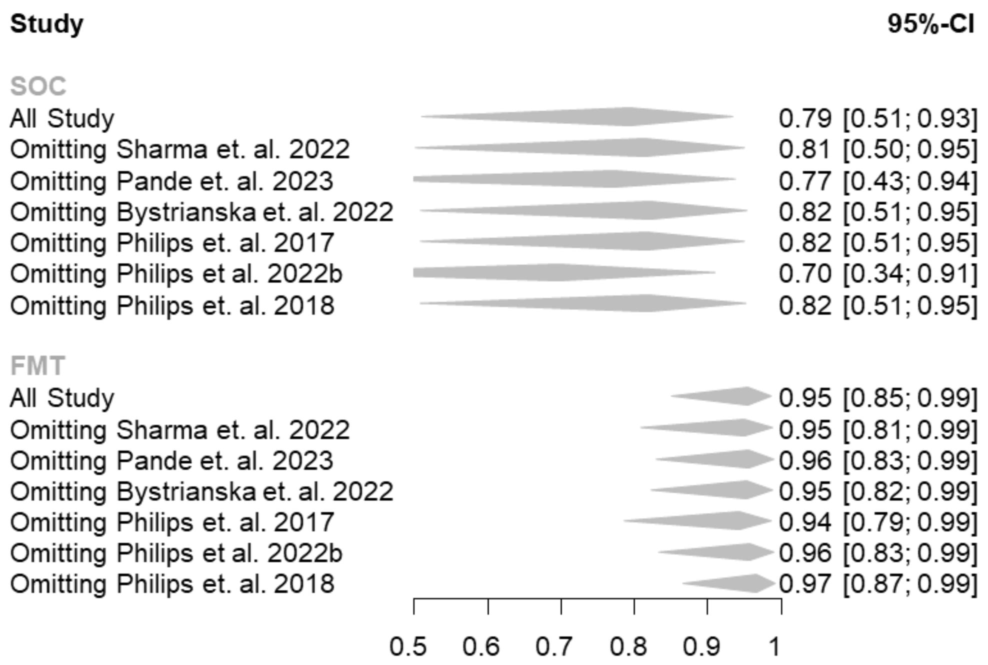

Supplementary Figure S14. Leave-one-out analysis of the 28-day survival probability for faecal microbiota transplantation vs standard of care.

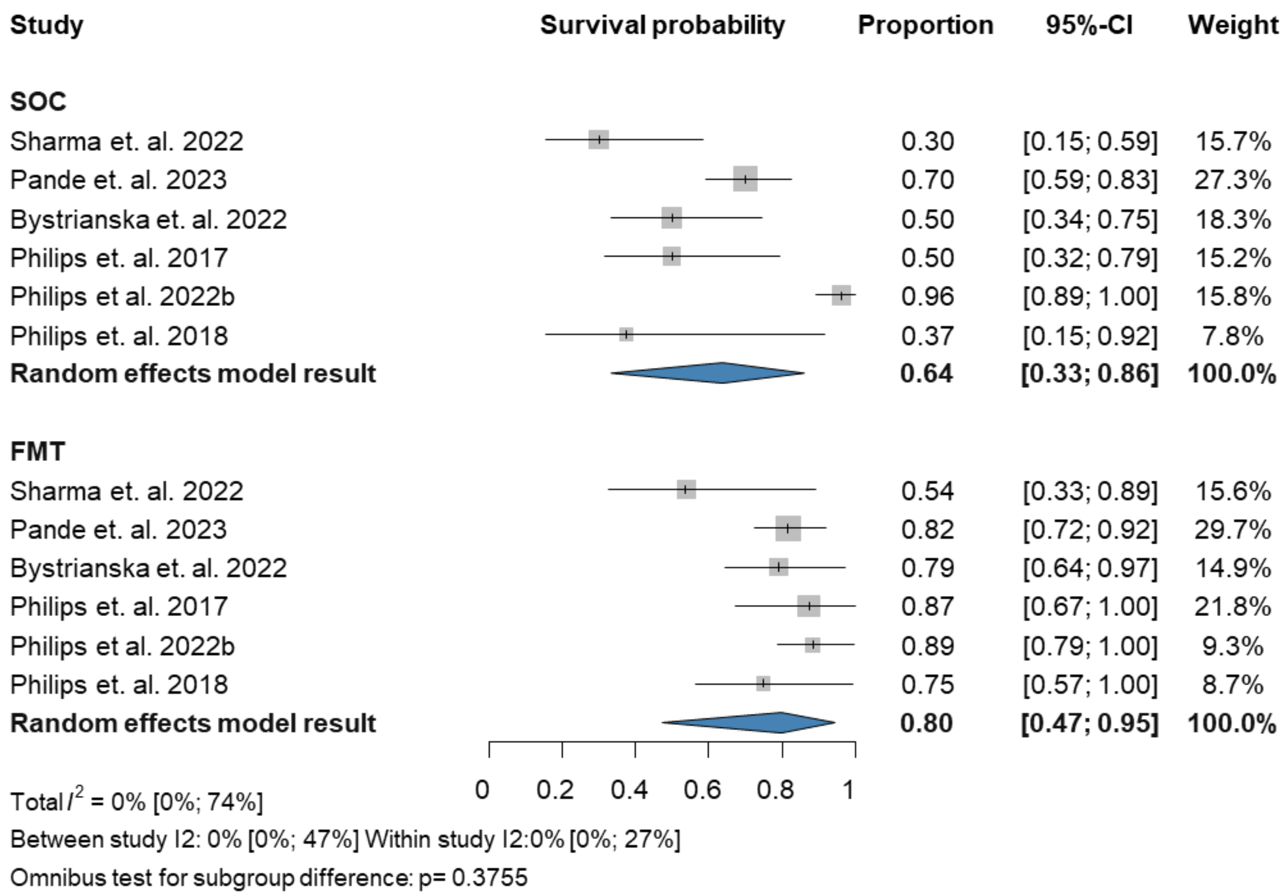

Supplementary Figure S15. Forest plot depicting the 90-day survival probability for faecal microbiota transplantation vs standard of care.

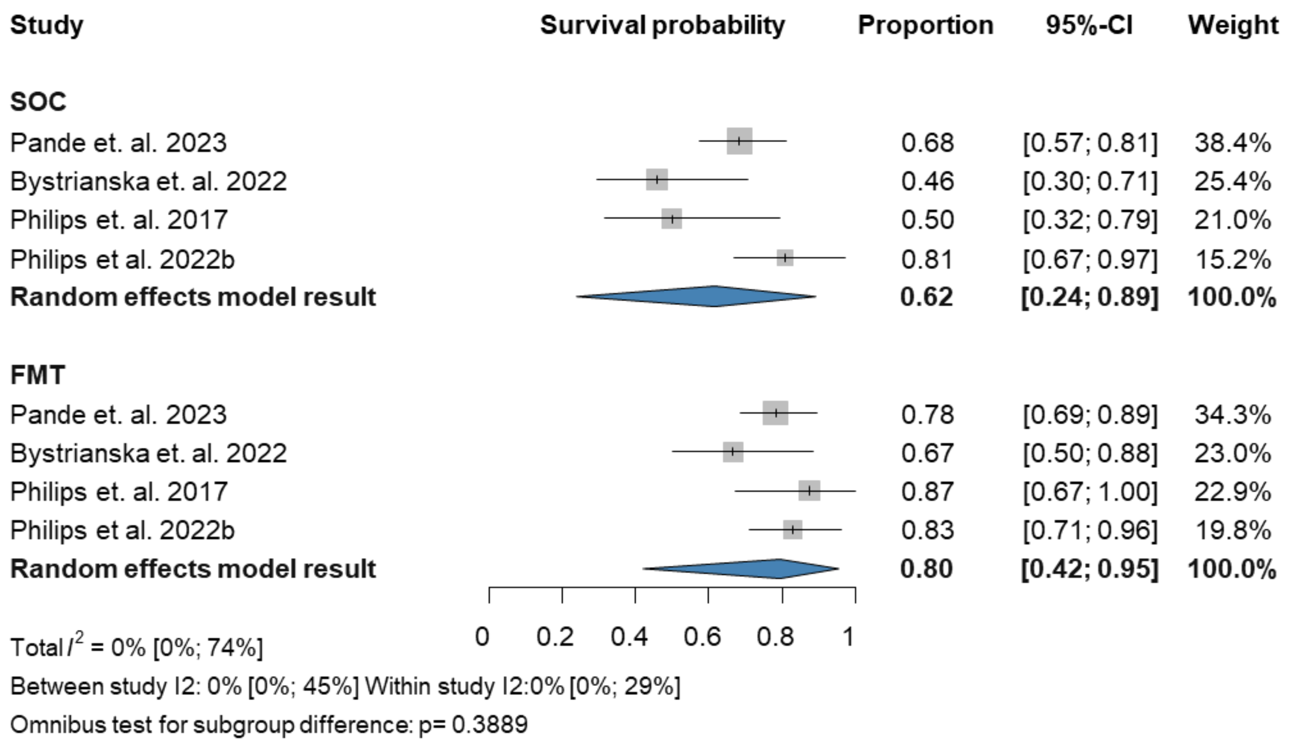

Supplementary Figure S16. Forest plot depicting the 180-day survival probability for faecal microbiota transplantation vs standard of care.

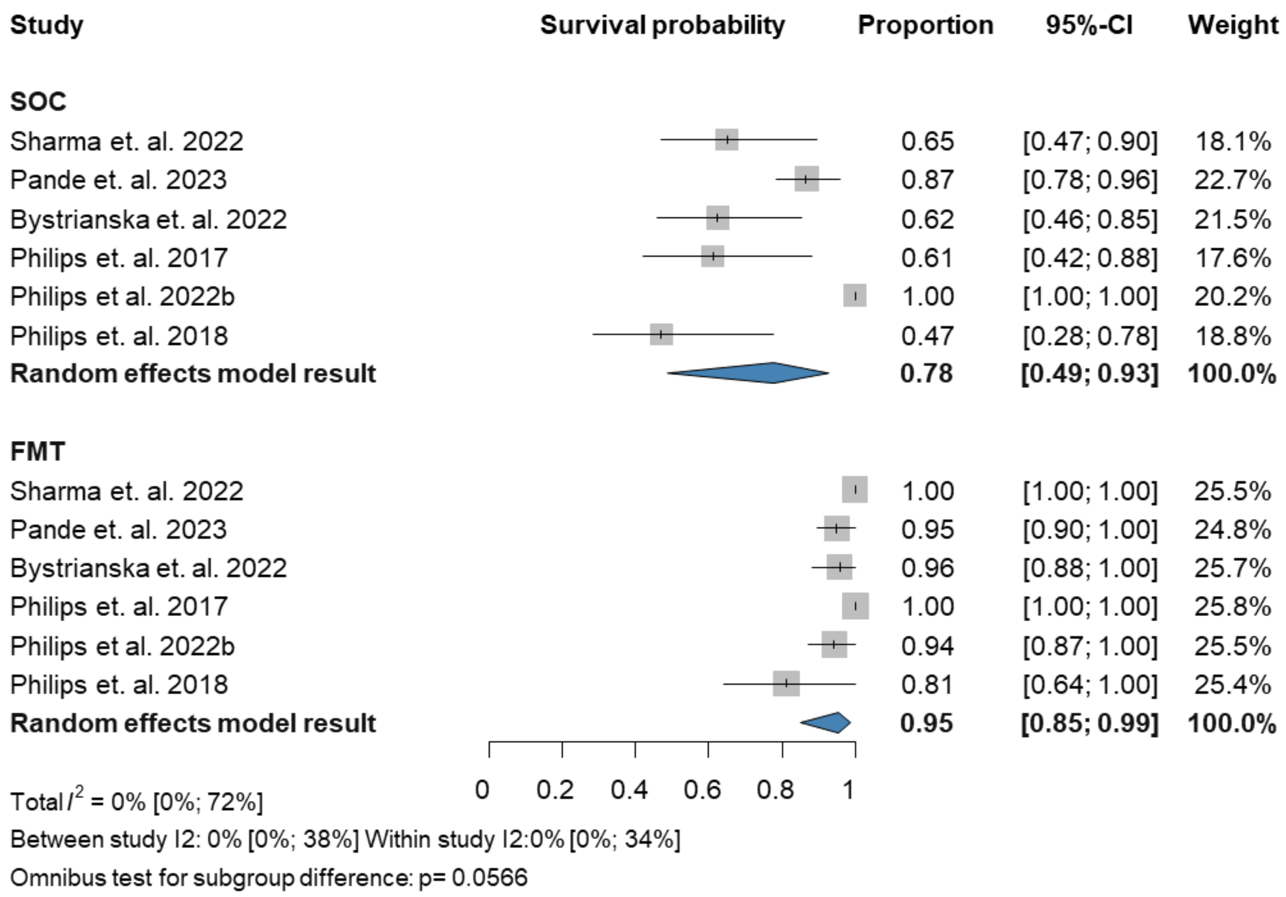

Supplementary Figure S17. Forest plot depicting the 28-day survival probability for faecal microbiota transplantation vs standard of care in a model utilizing the nutritional arm in Philips et al. (2018).

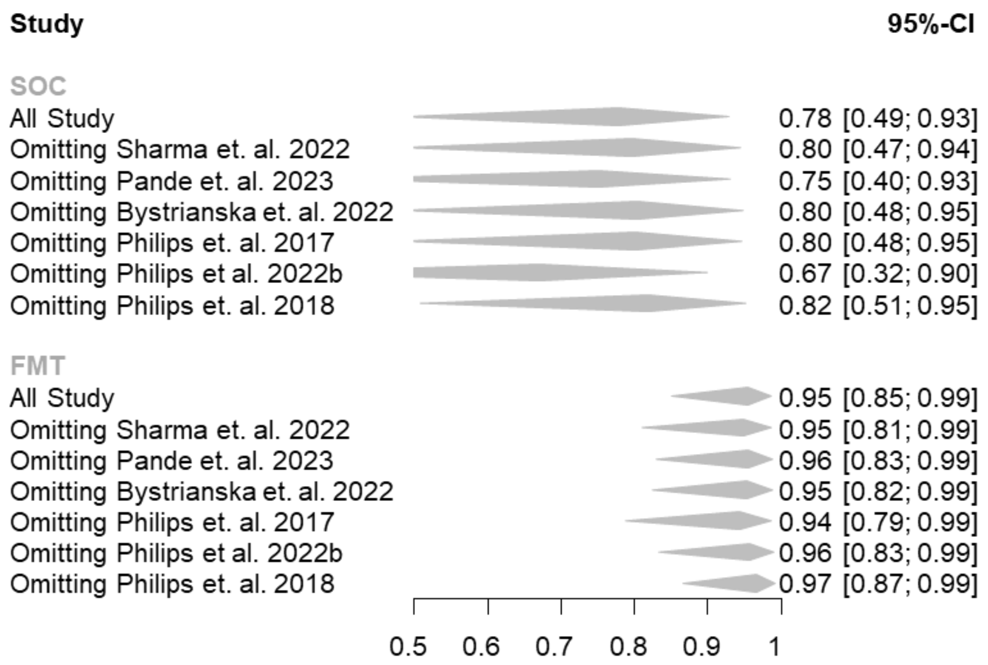

Supplementary Figure S18. Leave-one-out analysis of the 28-day survival probability for faecal microbiota transplantation vs standard of care in a model utilizing the nutritional arm in Philips et al. (2018).

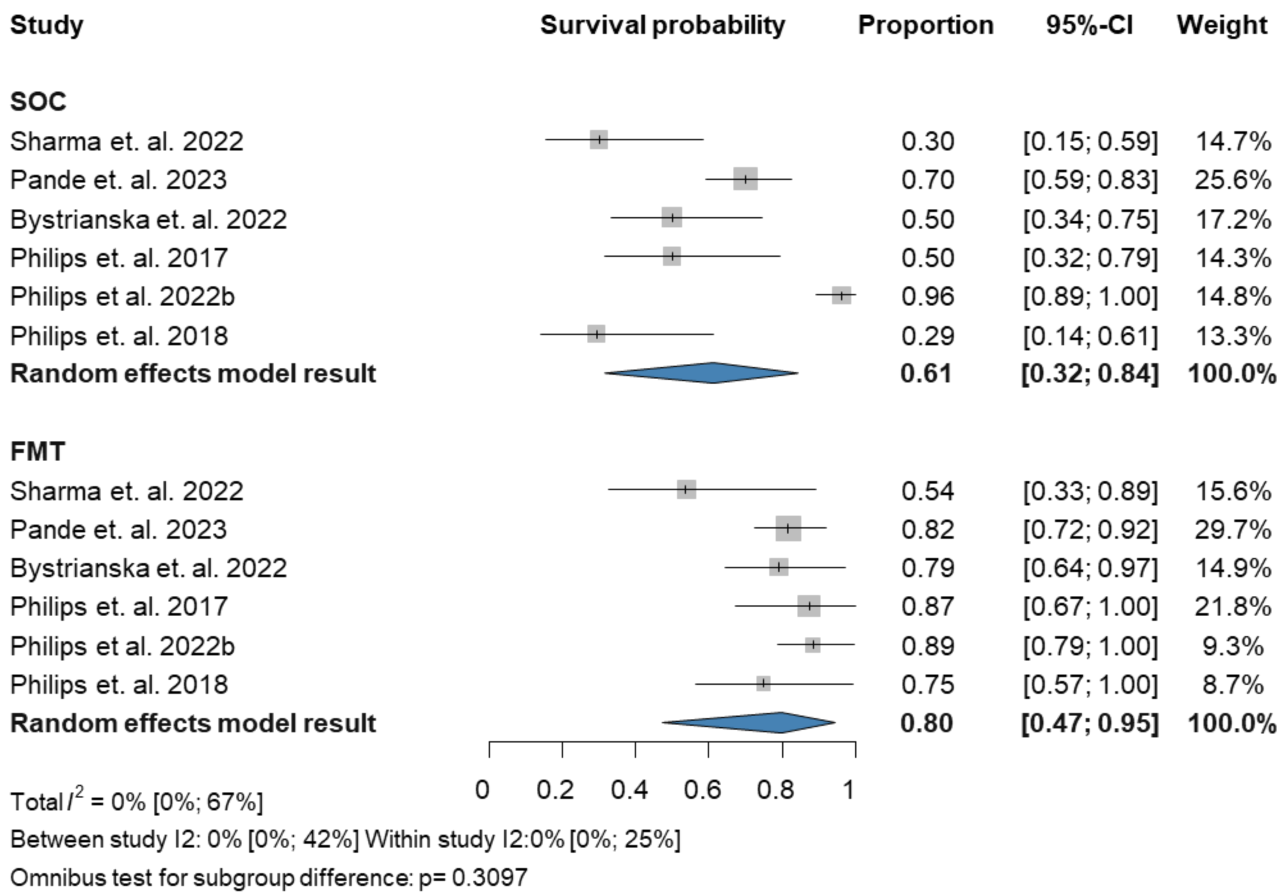

Supplementary Figure S19. Forest plot depicting the 90-day survival probability for faecal microbiota transplantation vs standard of care in model utilizing the nutritional arm in Philips et al. (2018).

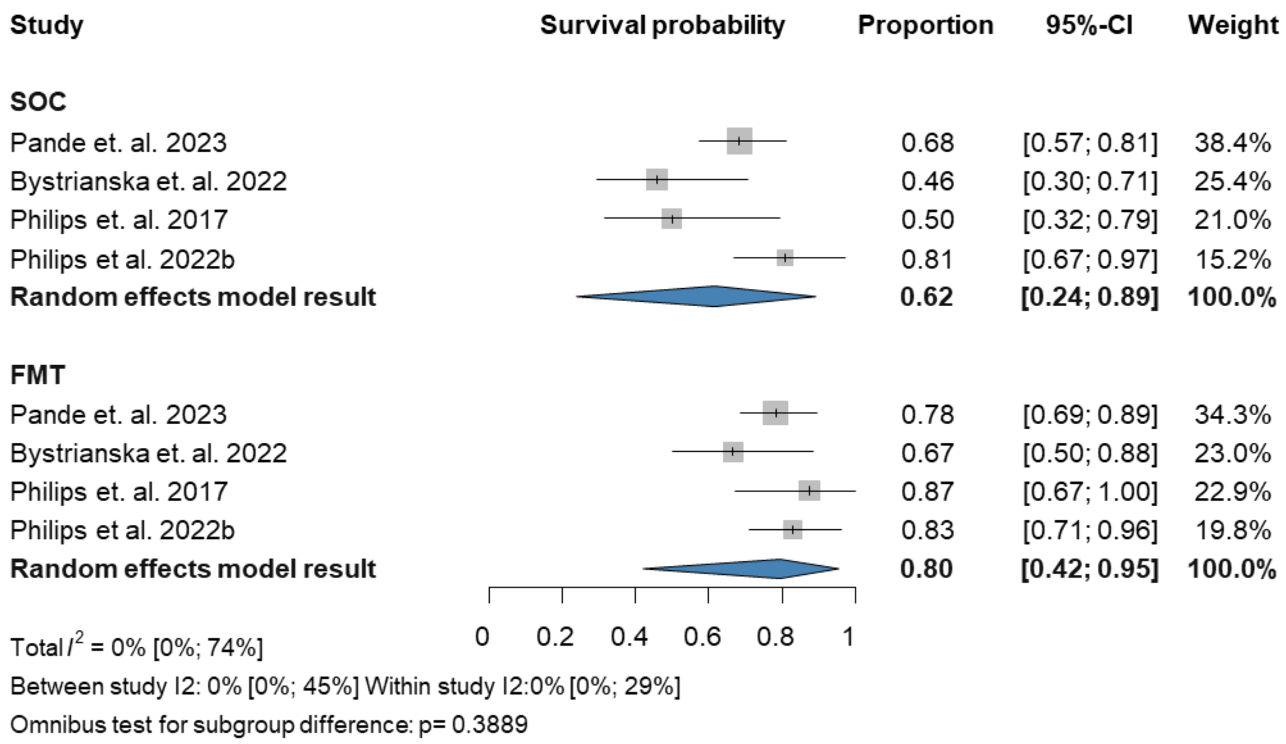

Supplementary Figure S20. Forest plot depicting the 180-day survival probability for faecal microbiota transplantation vs standard of care in a model utilizing the nutritional arm in Philips et al. (2018).

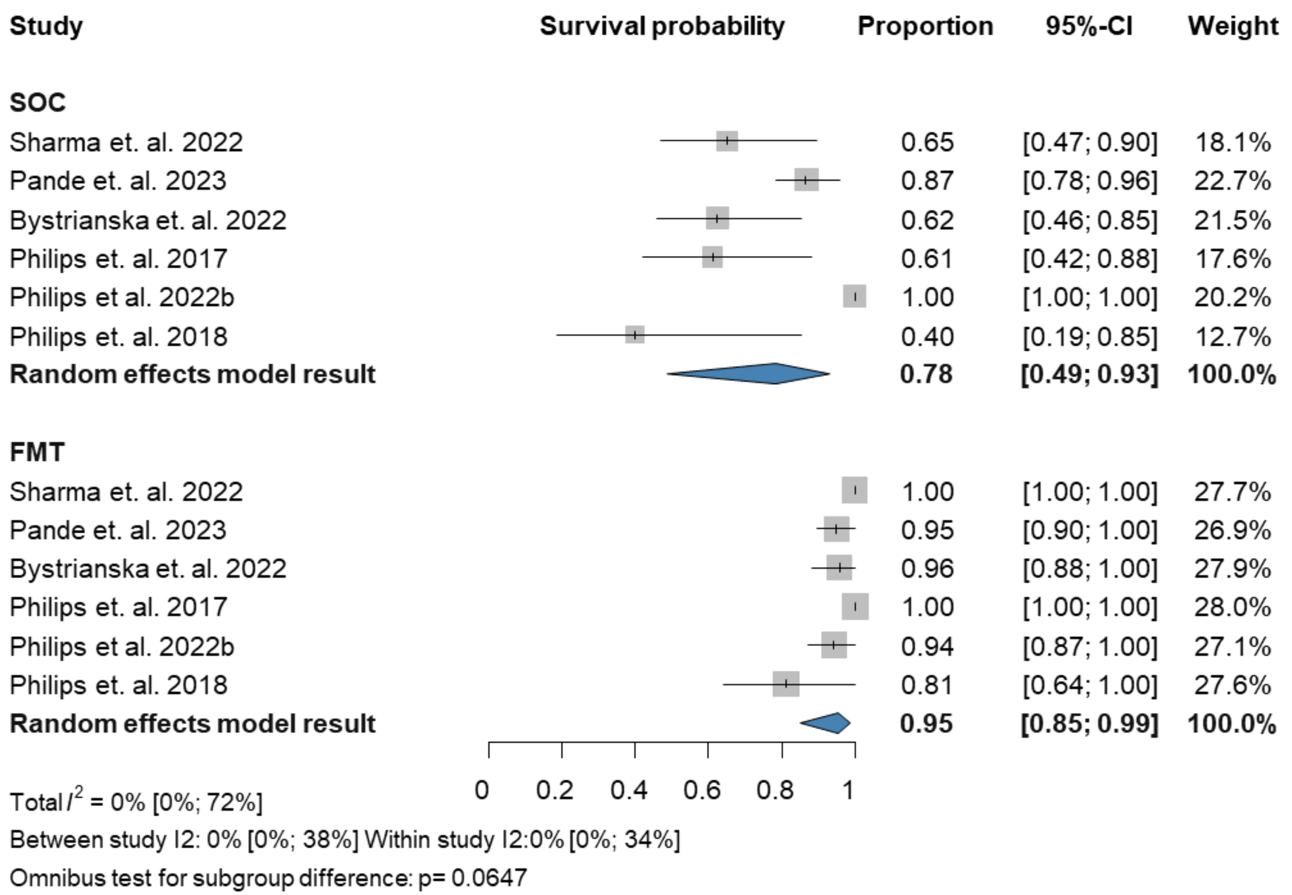

Supplementary Figure S21. Forest plot depicting the 28-day survival probability for faecal microbiota transplantation vs standard of care in a model utilizing the pentoxifylline arm in Philips et al. (2018).

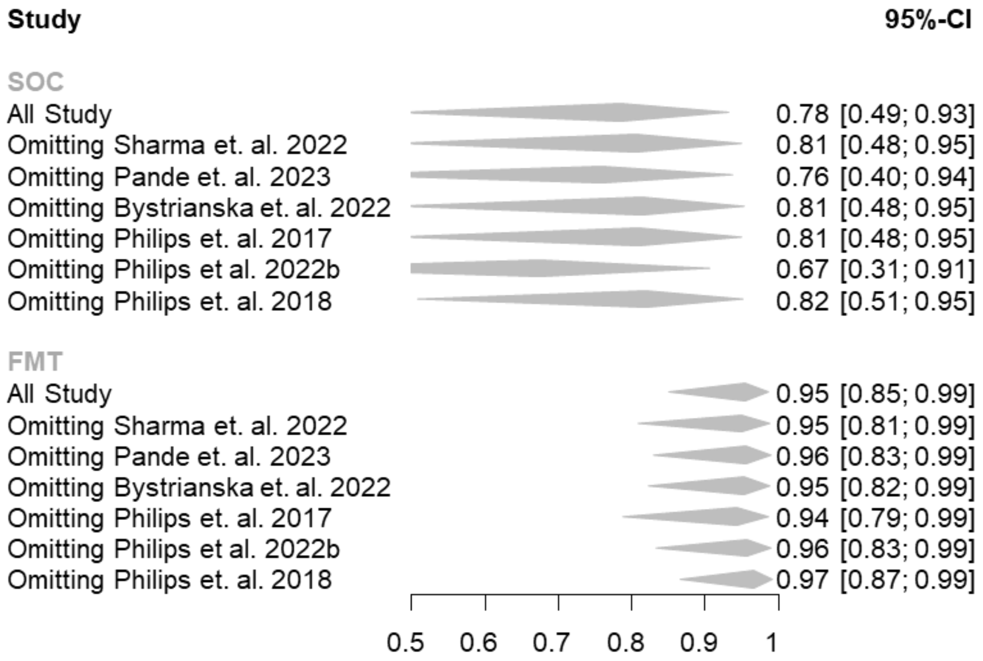

Supplementary Figure S22. Leave-one-out analysis of the 28-day survival probability for faecal microbiota transplantation vs standard of care in a model utilizing the pentoxifylline arm in Philips et al. (2018).

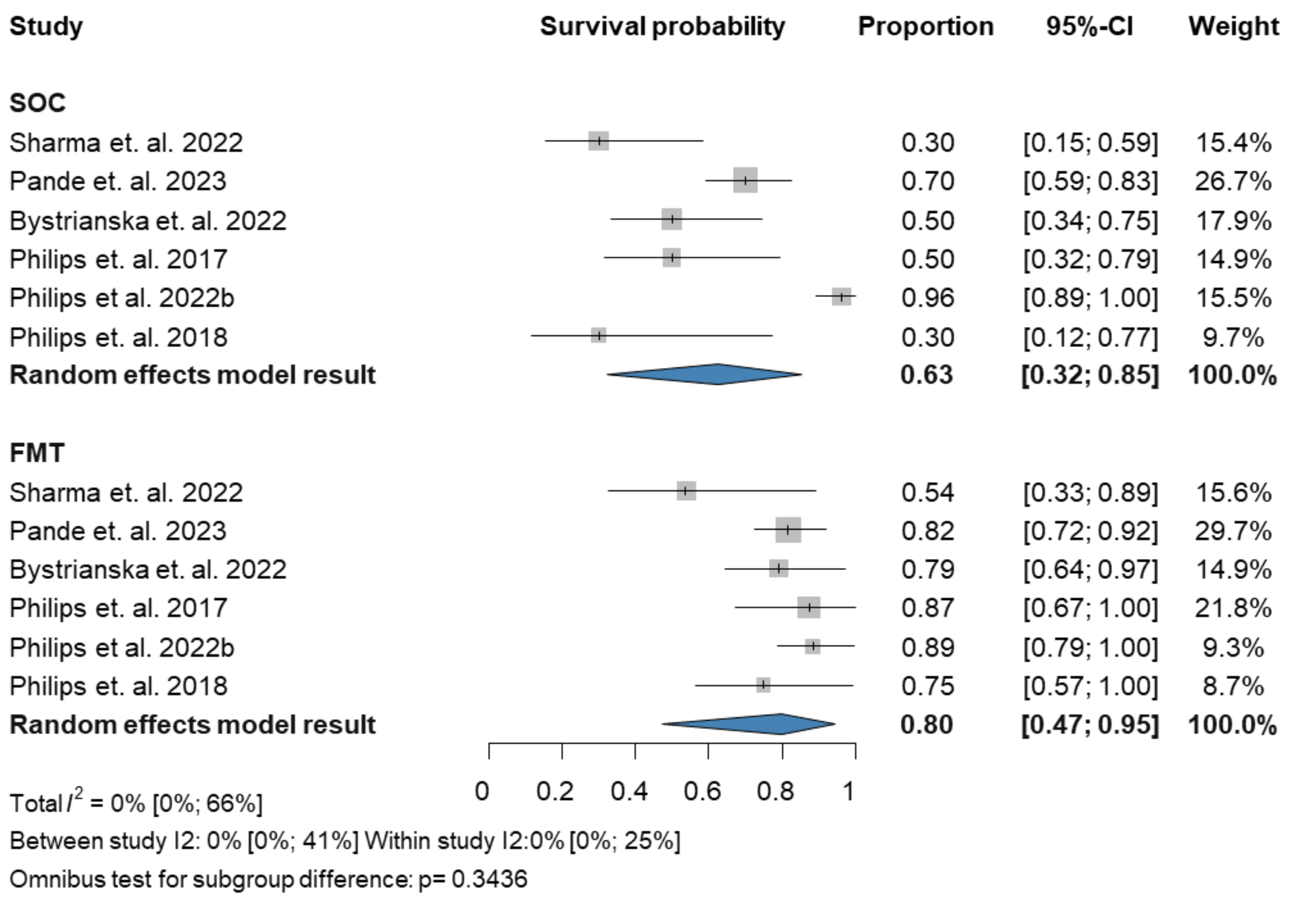

Supplementary Figure S23. Forest plot depicting the 90-day survival probability for faecal microbiota transplantation vs standard of care in a model utilizing the pentoxifylline arm in Philips et al. (2018).

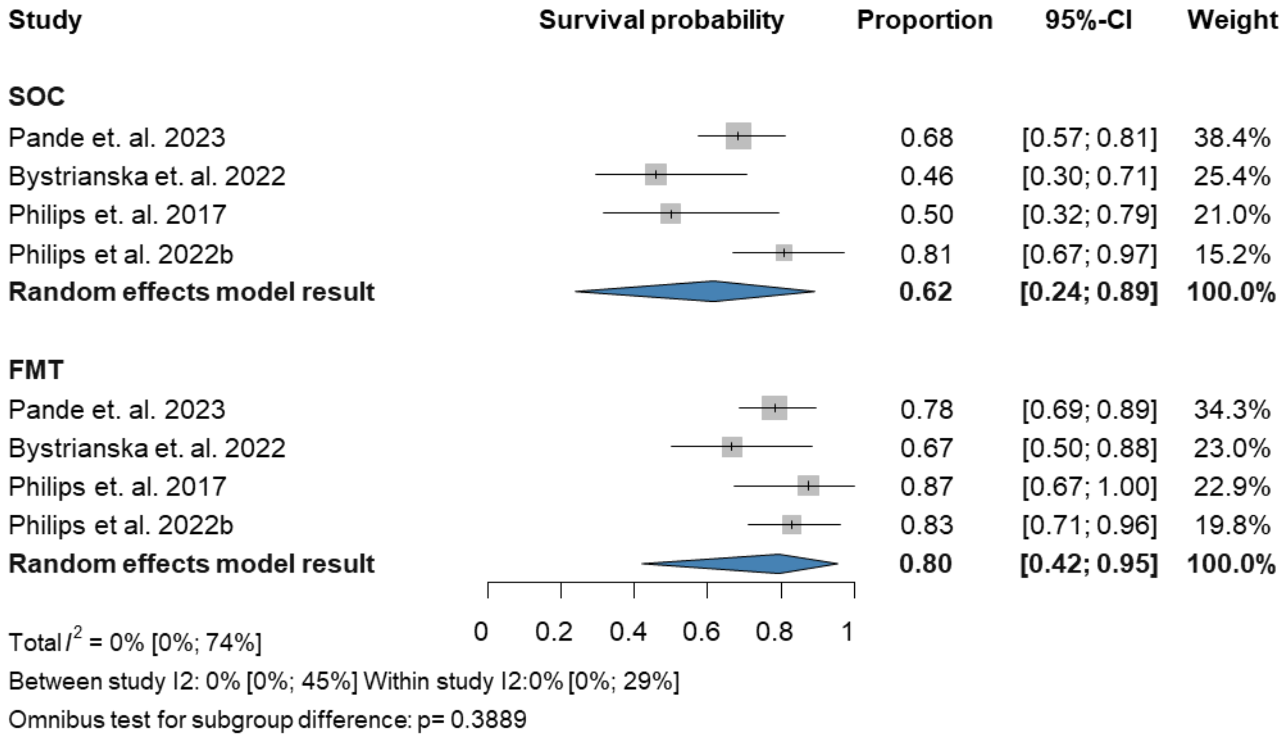

Supplementary Figure S24. Forest plot depicting the 180-day survival probability for faecal microbiota transplantation vs standard of care in a model utilizing the pentoxifylline arm in Philips et al. (2018).

Study	Comment	Experimental N	Experimental Mean	Experimental SD	Control N	Control Mean	Control SD	MD	95%-CI
Philips et al. 2018	PTX	16	25.80	3.8000	10	27.90	7.0000	-2.10	[-6.82; 2.62]
Bajaj et al. 2020	NA	10	12.00	2.9000	10	13.20	3.7000	-1.20	[-4.11; 1.71]
Sharma et al. 2022	NA	13	25.23	2.6500	20	25.55	3.0800	-0.32	[-2.29; 1.65]
Philips et al. 2018	CS	16	25.80	3.8000	8	26.00	3.3000	-0.20	[-3.15; 2.75]
Philips et al. 2022a	NA	47	28.10	4.7000	25	28.20	6.3000	-0.10	[-2.91; 2.71]
Pande et al. 2023	NA	60	25.33	2.7900	60	25.05	2.3200	0.28	[-0.64; 1.20]
Philips et al. 2022b	NA	35	26.60	4.1000	26	25.90	6.1300	0.70	[-2.02; 3.42]
Philips et al. 2018	NUT	16	25.80	3.8000	17	24.70	3.5000	1.10	[-1.40; 3.60]
Philips et al. 2017	NA	8	31.00	5.6000	18	27.00	5.2000	4.00	[-0.56; 8.56]

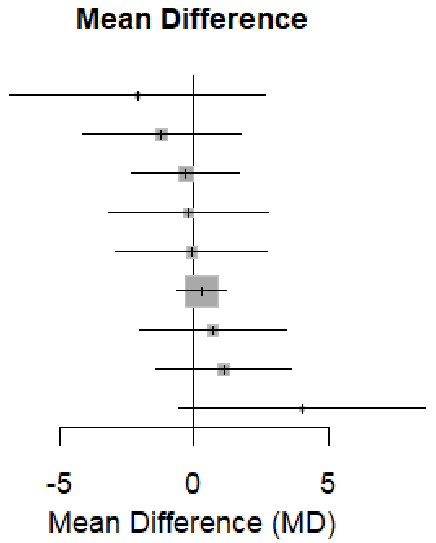

Supplementary Figure S25. Forest plot depicting the MD of MELD score at baseline between faecal microbiota transplantation vs standard of care arms of included studies.

MD: mean difference, MELD: model for End-Stage Liver Disease

Study	Comment	FMT N	FMT Mean	FMT SD	SOC N	SOC Mean	SOC SD	Mean Difference	MD	95% CI	Weight
Sharma et al. 2022	NA	13	11.46	1.27	20	12.05	1.05		-0.59	[-1.42; 0.24]	13.57%
Pande et al. 2023	NA	60	11.07	0.91	60	11.12	0.95		-0.05	[-0.38; 0.28]	16.39%
Philips et al. 2022b	NA	35	11.80	1.59	26	11.20	1.75		0.60	[-0.25; 1.45]	13.38%
Philips et al. 2018	Nut	16	13.20	0.70	17	12.50	1.10		0.70	[0.07; 1.33]	14.88%
Philips et al. 2018	CS	16	13.20	0.70	8	12.30	1.30		0.90	[-0.06; 1.86]	12.64%
Philips et al. 2018	PTX	16	13.20	0.70	10	12.30	0.90		0.90	[0.25; 1.55]	14.69%
Philips et al. 2017	NA	8	14.00	0.80	18	11.70	0.90		2.30	[1.61; 2.99]	14.45%

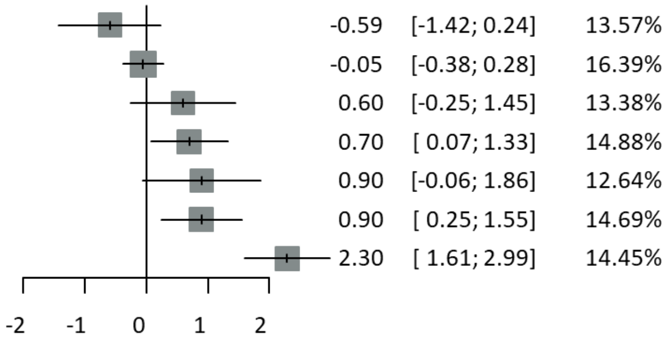

Supplementary Figure S26. Forest plot depicting the MD of CPT score at baseline between FMT and SOC arm in included studies.

CPT: Child–Pugh–Turcotte, MD: mean difference

Study	Comment	FMT N	FMT Mean	FMT SD	SOC N	SOC Mean	SOC SD	Mean Difference	MD	95% CI	Weight
Philips et al. 2018	PTX	16	73.90	14.90	10	90.90	36.00		-17.00	[-40.48; 6.48]	4.46%
Philips et al. 2018	Nut	16	73.90	14.90	17	79.20	31.70		-5.30	[-22.04; 11.44]	8.73%
Philips et al. 2018	CS	16	73.90	14.90	8	77.80	18.90		-3.90	[-18.89; 11.09]	10.88%
Philips et al. 2022b	NA	35	95.30	30.30	26	95.10	38.52		0.20	[-17.69; 18.09]	7.66%
Pande et al. 2023	NA	60	68.13	15.96	60	65.10	18.28		3.03	[-3.11; 9.17]	62.63%
Sharma et al. 2022	NA	13	87.00	29.01	20	83.59	31.16		3.41	[-17.45; 24.27]	5.64%

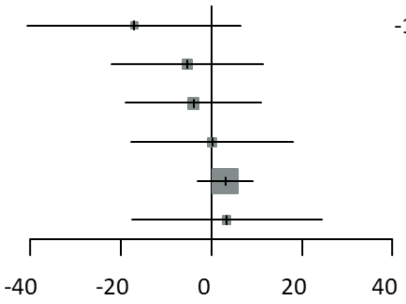

Supplementary Figure S27. Forest plot depicting the MD of MDF score at baseline between FMT and SOC arm in included studies.

MD: mean difference; MDF: Maddrey's Discriminant Function

Table & Legends:

Section and Topic	Item #	Checklist item	Location where item is reported
**TITLE**			
Title	1	Identify the report as a systematic review.	Page 1
**ABSTRACT**			
Abstract	2	See the PRISMA 2020 for Abstracts checklist.	Page 3
**INTRODUCTION**			
Rationale	3	Describe the rationale for the review in the context of existing knowledge.	Page 4
Objectives	4	Provide an explicit statement of the objective(s) or question(s) the review addresses.	Page 4
**METHODS**			
Eligibility criteria	5	Specify the inclusion and exclusion criteria for the review and how studies were grouped for the syntheses.	Page 5
Information sources	6	Specify all databases, registers, websites, organisations, reference lists and other sources searched or consulted to identify studies. Specify the date when each source was last searched or consulted.	Page 5
Search strategy	7	Present the full search strategies for all databases, registers and websites, including any filters and limits used.	Suppl. material
Selection process	8	Specify the methods used to decide whether a study met the inclusion criteria of the review, including how many reviewers screened each record and each report retrieved, whether they worked independently, and if applicable, details of automation tools used in the process.	Page 5
Data collection process	9	Specify the methods used to collect data from reports, including how many reviewers collected data from each report, whether they worked independently, any processes for obtaining or confirming data from study investigators, and if applicable, details of automation tools used in the process.	Page 5
Data items	10a	List and define all outcomes for which data were sought. Specify whether all results that were compatible with each outcome domain in each study were sought (e.g. for all measures, time points, analyses), and if not, the methods used to decide which results to collect.	Page 5
	10b	List and define all other variables for which data were sought (e.g. participant and intervention characteristics, funding sources). Describe any assumptions made about any missing or unclear information.	Page 5
Study risk of bias assessment	11	Specify the methods used to assess risk of bias in the included studies, including details of the tool(s) used, how many reviewers assessed each study and whether they worked independently, and if applicable, details of automation tools used in the process.	Page 5
Effect measures	12	Specify for each outcome the effect measure(s) (e.g. risk ratio, mean difference) used in the synthesis or presentation of results.	Page 6
Synthesis methods	13a	Describe the processes used to decide which studies were eligible for each synthesis (e.g. tabulating the study intervention characteristics and comparing against the planned groups for each synthesis (item #5)).	Page 6 + Supp material
	13b	Describe any methods required to prepare the data for presentation or synthesis, such as handling of missing summary statistics, or data conversions.	Page 6 + Supp material
	13c	Describe any methods used to tabulate or visually display results of individual studies and syntheses.	Page 6 + Supp material

Section and Topic	Item #	Checklist item	Location where item is reported
	13d	Describe any methods used to synthesize results and provide a rationale for the choice(s). If meta-analysis was performed, describe the model(s), method(s) to identify the presence and extent of statistical heterogeneity, and software package(s) used.	Page 6 + Supp material
	13e	Describe any methods used to explore possible causes of heterogeneity among study results (e.g. subgroup analysis, meta-regression).	Page 5
	13f	Describe any sensitivity analyses conducted to assess robustness of the synthesized results.	Page 6 + Supp material
Reporting bias assessment	14	Describe any methods used to assess risk of bias due to missing results in a synthesis (arising from reporting biases).	Page 6 + Supp material
Certainty assessment	15	Describe any methods used to assess certainty (or confidence) in the body of evidence for an outcome.	Page 6 + Supp material
**RESULTS**			
Study selection	16a	Describe the results of the search and selection process, from the number of records identified in the search to the number of studies included in the review, ideally using a flow diagram.	Page 6
	16b	Cite studies that might appear to meet the inclusion criteria, but which were excluded, and explain why they were excluded.	Page 6
Study characteristics	17	Cite each included study and present its characteristics.	Page 7 and Table 1
Risk of bias in studies	18	Present assessments of risk of bias for each included study.	Page 10 and Figure 4 and Suppl figure 4-6
Results of individual studies	19	For all outcomes, present, for each study: (a) summary statistics for each group (where appropriate) and (b) an effect estimate and its precision (e.g. confidence/credible interval), ideally using structured tables or plots.	Page 7-9
Results of syntheses	20a	For each synthesis, briefly summarise the characteristics and risk of bias among contributing studies.	Page 10 and Figure 4 and Suppl figure 4-6
	20b	Present results of all statistical syntheses conducted. If meta-analysis was done, present for each the summary estimate and its precision (e.g. confidence/credible interval) and measures of statistical heterogeneity. If comparing groups, describe the direction of the effect.	Page 3-4
	20c	Present results of all investigations of possible causes of heterogeneity among study results.	Page 8,9
	20d	Present results of all sensitivity analyses conducted to assess the robustness of the synthesized results.	Page 12+ Supp figure 2,6,11.14.18.22
Reporting biases	21	Present assessments of risk of bias due to missing results (arising from reporting biases) for each synthesis assessed.	Supp. Figure 6-9
Certainty of evidence	22	Present assessments of certainty (or confidence) in the body of evidence for each outcome assessed.	Page 7-8 and Supp. table 10

Section and Topic	Item #	Checklist item	Location where item is reported
**DISCUSSION**			
Discussion	23a	Provide a general interpretation of the results in the context of other evidence.	Page 12-14
	23b	Discuss any limitations of the evidence included in the review.	Page 13
	23c	Discuss any limitations of the review processes used.	Page 13
	23d	Discuss implications of the results for practice, policy, and future research.	Page 13-14
**OTHER INFORMATION**			
Registration and protocol	24a	Provide registration information for the review, including register name and registration number, or state that the review was not registered.	Page 5
	24b	Indicate where the review protocol can be accessed, or state that a protocol was not prepared.	Page 5
	24c	Describe and explain any amendments to information provided at registration or in the protocol.	Page 5
Support	25	Describe sources of financial or non-financial support for the review, and the role of the funders or sponsors in the review.	Page 2
Competing interests	26	Declare any competing interests of review authors.	Page 2
Availability of data, code and other materials	27	Report which of the following are publicly available and where they can be found: template data collection forms; data extracted from included studies; data used for all analyses; analytic code; any other materials used in the review.	Page 2

From: Page MJ, McKenzie JE, Bossuyt PM, Boutron I, Hoffmann TC, Mulrow CD, et al. The PRISMA 2020 statement: an updated guideline for reporting systematic reviews. BMJ 2021;372:n71. doi: 10.1136/bmj.n71. This work is licensed under CC BY 4.0. To view a copy of this license, visit https://creativecommons.org/licenses/by/4.0/

Supplementary Table S1. PRISMA checklist

	FMT	CS	PTX	NUT
FMT	-	1,54<br>(95CI 0.78 - 3.03)<br>p 0.36	**2.22**<br>**(95CI 1.04 – 4.76)**<br>**p 0.03**	**2.78**<br>**(95CI1.52 -5.26)**<br>**p<0.01**
CS	0.65<br>(95CI 0.33 - 1.28)<br>p 0.36	-	1.43<br>(95CI 0.55 -3.70)<br>p0.77	1.82<br>(95CI 0.79 – 4.17)<br>p 0.25
PTX	**0.45**<br>**(95CI 0.21 - 0.96)**<br>**p 0.03**	0.70<br>(95CI 0.27 - 1.82)<br>p 0.77	-	1.28<br>(95CI 0.54 - 3.03)<br>p 0.89
NUT	**0.36**<br>**(95CI 0.19 - 0.66)**<br>**p<0.01**	0.55<br>(95CI 0.24 - 1.27)<br>p 0.25	0.78<br>(95CI 0.33 -1.85)<br>p 0.89	-

Supplementary Table S2: Pairwise Comparisons of HRs for All Treatment Arms with Tukey Multiplicity Corrected p-values and CI (Calculated on Log-Scale)

95CI: 95% Confidence Interval, CS: Glucocorticoids, FMT: Fecal Microbiota Transplantation, HR: Hazard Ratio, NUT: Nutritional Support Only, PTX: Pentoxifylline

Study	Sequencing method	Comparator groups	Time of evolution	Evaluation method
Pande et. al. (2023)	16S rRNA sequencing (V3–V4)	FMT+SOC vs Donors<br>FMT vs SOC<br>Temporal analysis	At baseline<br>28,90 days	Alpha diversity (Shannon,Chao1, Simpson index)<br>Fold change between each taxon<br>Principal component analysis<br>Cox regression analysis(taxa association with mortality)
Philips et. al. (2018)	16S rRNA sequencing (V3–V4)	FMT vs healthy control<br>Temporal analysis	At baseline.<br>8, 30, 90 days	Relative abundance<br>Linear discriminant analysis effect size<br>Functional microbial pathways
Philips et al. (2022a)	16S rRNA sequencing (V3–V4)	FMT vs SOC	30,180 days	Linear discriminant analysis effect size<br>Network analysis
Philips et al. (2022b)	16S rRNA sequencing (V3–V4)	FMT vs SOC<br>Temporal analysis	At baseline<br>follow-up to 1-2 years and 3-4 years	Relative abundance<br>Linear discriminant analysis<br>Linear discriminant analysis effect size
Philips et. al. (2023)	16S rRNA sequencing (V3–V4)	FMT vs SOC<br>Daily vs binge drinking patterns<br>alcohol relapsers vs no-relapsers	Baseline	Relative abundance<br>Alpha diversity (Shannon index)<br>Beta diversity (Bray-Curtis)<br>Linear discriminant analysis<br>Principal component analysis (on beta diversity)<br>Linear Discriminant Analysis Effect Size<br>Functional microbial pathways
Philips et. al (2017)	NA	FMT vs Donors	At baseline<br>180, 360 days	Relative abundance<br>Functional microbial pathways

Supplementary Table S3. Overview of the microbiota data reported in the included studies

FMT: Faecal Microbiota Transplantation, SOC: Standard of Care

Author (Year)	Severity score	Baseline (Mean±SE)		Follow up time						Difference	
				7 (Days)		28 (Days)		90 (Days)			
		FMT	SOC	FMT	SOC	FMT	SOC	FMT	SOC	FMT	SOC
Sharma et. al.(2022)	MELD	25.2 ±0.73	25.55 ±0.69	22.7 ±1.32	25.5 ±0.64	22 ±1.80	24.5 ±1.84	12.71 ±1.89	16.2 ±2.82	- 12.49 ±2.03	- 9.35 ±2.9
Sharma et. al.(2022)	CPT	11.46 ±0.35	12.05 ±0.23	10.38 ±0.27	11.85 ±0.28	9.92 ±0.51	10.58 ±0.56	6.57 ±0.64	7.6 ±0.68	- 4.89 ±0.73	- 4.45 ±0.72
Sharma et. al.(2022)	MDF	87 ±8.03	83.59 ±6.96	76.8 ±10.23	87.53 ±8.03	66.93 ±12.78	80.9 ±15.68	22.2 ±7.71	25.54 ±11.36	- 64.8 ±11.13	- 58.05 ±13.33
Pande et. al.(2023)	MELD	25.33 ±0.36	25.05 ±0.30	NA	NA	24.75 ±0.86	22.96 ±0.98	21.13 ±1.05	21.88 ±1.23	- 4.20 ±1.0	- 3.17 ±1.15
Pande et. al.(2023)	CPT	11.09 ±0.12	11.18 ±0.12	NA	NA	10.96 ±0.14	10.67 ±0.26	10.07 ±0.19	9.80 ±0.32	- 1.02 ±0.17	- 1.37 ±0.27
Pande et. al.(2023)	MDF	68.13 ±2.06	65.1 ±2.36	NA	NA	69.51 ±5.30	58.60 ±5.81	60.70 ±8.40	56.76 ±6.56	- 7.43 ±8.23	- 8.33 ±6.40
Philips et. al(2017)	MELD	31 ±1.98	27 ±1.23	NA	NA	NA	NA	12.3 ±1.31	NA	- 18.7 ±2.4	NA
Philips et. al(2017)	CPT	14.5 ±0.28	11.7 ±0.21	NA	NA	NA	NA	7.7 ±0.42	NA	- 6.8 ±0.5	NA
Bajaj et. al. (2020)	MELD	12 ±2.9*	13.2 ±3.7*	NA	NA	NA	NA	NA	NA	- 0.1 ±2*	- 0.2 ±2*

Supplementary Table S4. Severity Score Comparisons at Baseline during Follow-ups and difference between baseline and end of follow up

*CPT: Child–Pug–Turcotte h, FMT: Fecal Microbiota Transplantation, MELD: Model for End-Stage Liver Disease, MDF: Maddrey Discriminant Function, SD: Standard Deviation, SE: Standard Error, SOC: Standard of Care, *mean and SD*

	**Ascites**				**Hepatic encephalopathy**				**Infections**				**Spontaneous bacterial peritonitis**				**Excessive flatulence**	
	**Baseline**		**Follow-up**		**Baseline**		**Follow-up**		**Baseline**		**Follow-up**		**Baseline**		**Follow-up**		**During Hospitalization**	
**Author**<br>**(year)**	**FMT**<br>**N0 (%)**	**SOC**<br>**N0 (%)**	**FMT**<br>**N0 (%)**	**SOC**<br>**N0 (%)**	**FMT**<br>**N0 (%)**	**SOC**<br>**N0 (%)**	**FMT**<br>**N0 (%)**	**SOC**<br>**N0 (%)**	**FMT**<br>**N0 (%)**	**SOC**<br>**N0 (%)**	**FMT**<br>**N0 (%)**	**SOC**<br>**N0 (%)**	**FMT**<br>**N0 (%)**	**SOC**<br>**N0 (%)**	**FMT**<br>**N0 (%)**	**SOC**<br>**N0 (%)**	**FMT**<br>**N0 (%)**	**SOC**<br>**N0 (%)**
Sharma et. al.(2022)	**13**<br>**(100%)**	**20**<br>**(100%)**	**0**<br>**(0%)**	**12**<br>**(60%)**	**6**<br>**(46.2%)**	**14**<br>**(70%)**	**0**<br>**(0%)**	**4**<br>**(20%)**	**3**<br>**(23%)**	**4**<br>**(20%)**	NA	NA	**4**<br>**(30,1%)**	**5**<br>**(25%)**	**3**<br>**(23%)**	**2**<br>**(10%)**	**13**<br>**(100%)**	**1**<br>**(5%)**
Pande et. al.(2023)	NA	NA	**0**<br>**(0%)**	**4**<br>**(7.0%)**	NA	NA	NA	NA	NA	NA	**10**<br>**(18.2%)**	**16**<br>**(28.7)**	NA	NA	**1**<br>**(1.8%)**	**1**<br>**(1.8%)**	**4**<br>**(7,2%)**	**2**<br>**(3,5%)**
Philips et. al. (2018)	**16**<br>**(100%)**	**28**<br>**(80%)**	**0**<br>**(0%)**	**1**<br>**(2.8%)**	**16**<br>**(100%)**	**35**<br>**(100%)**	**0**<br>**(0%)**	**11**<br>**(32%)**	NA	NA	NA	NA	NA	NA	NA	NA	NA	NA
Philips et al. 2022a)	**31**<br>**(65,9%)**	**18**<br>**(72.0%)**	**12**<br>**(25,5%)**	**14**<br>**(56.0%)**	**30**<br>**(63,8%)**	**15**<br>**(66,0%)**	**5**<br>**(10,6%)**	**10**<br>**(40%)**	**16**<br>**(34%)**	**11**<br>**(44%)**	**7**<br>**(14.8%)**	**11**<br>**(44%)**	NA	NA	NA	NA	**12**<br>**(25,5%)**	**0**<br>**(0%)**
Philips et al. 2022b)	**23**<br>**(66%)**	**15**<br>**(58%)**	**12**<br>**(34%)**	**19**<br>**(73%)**	**20**<br>**(57.1%)**	**11**<br>**(42.3%)**	**7**<br>**(20%)**	**18**<br>**(69.2%)**	**17**<br>**(48.5%)**	**6**<br>**(23.0%)**	**6**<br>**(17.1%)**	**14**<br>**(53,8%)**	NA	NA	**2**<br>**(5.7%)**	**4**<br>**(15.3%)**	NA	NA
Bajaj et. al. (2020)	NA	NA	NA	NA	NA	NA	NA	NA	**0**<br>**(0%)**	**2**<br>**(20%)**	NA	NA	NA	NA	NA	NA	NA	NA
SUM	83	81	24	50	72	75	12	43	**36**	**23**	23	41	4	5	6	7	29	3

Supplementary Table S5. The adverse event rate at base line and follow up

FMT: Faecal Microbiota Transplantation, SOC: Standard of Care

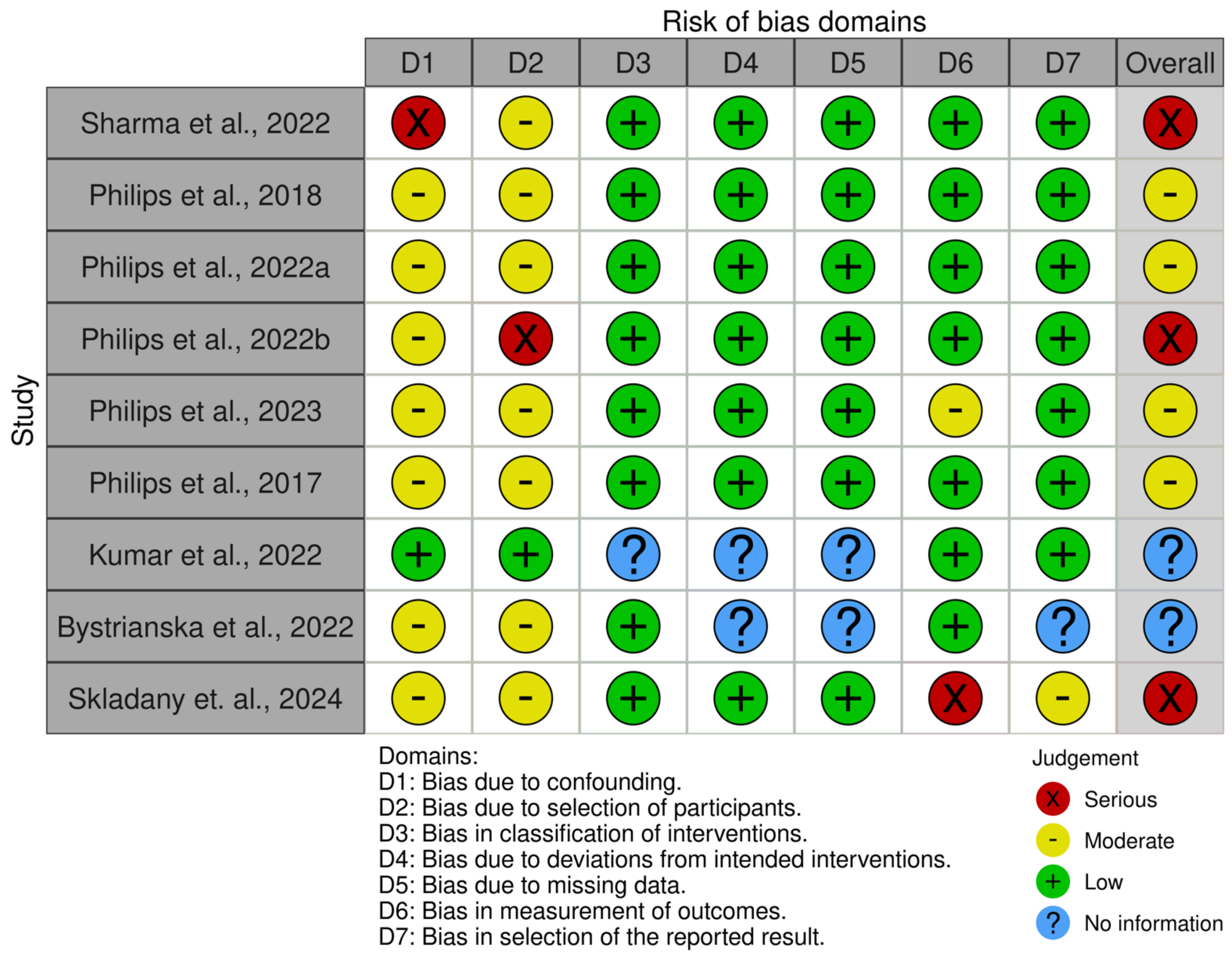
Risk of bias domains
D1 D2 D3 D4 D5 D6 D7 Overall
Study
Sharma et al., 2022 X - + + + + + X
Philips et al., 2018 - - + + + + + -
Philips et al., 2022a - - + + + + + -
Philips et al., 2022b - X + + + + + X
Philips et al., 2023 - - + + + - + -
Philips et al., 2017 - - + + + + + -
Kumar et al., 2022 + + ? ? ? + + ?
Bystrianska et al., 2022 - - + ? ? + ? ?
Skladany et. al., 2024 - - + + + X - X
Domains:
D1: Bias due to confounding.
D2: Bias due to selection of participants.
D3: Bias in classification of interventions.
D4: Bias due to deviations from intended interventions.
D5: Bias due to missing data.
D6: Bias in measurement of outcomes.
D7: Bias in selection of the reported result.
Judgement
X Serious
- Moderate
+ Low
? No information

Supplementary Table S6. Robvis traffic light plot depicting the risk of bias in non-randomized studies

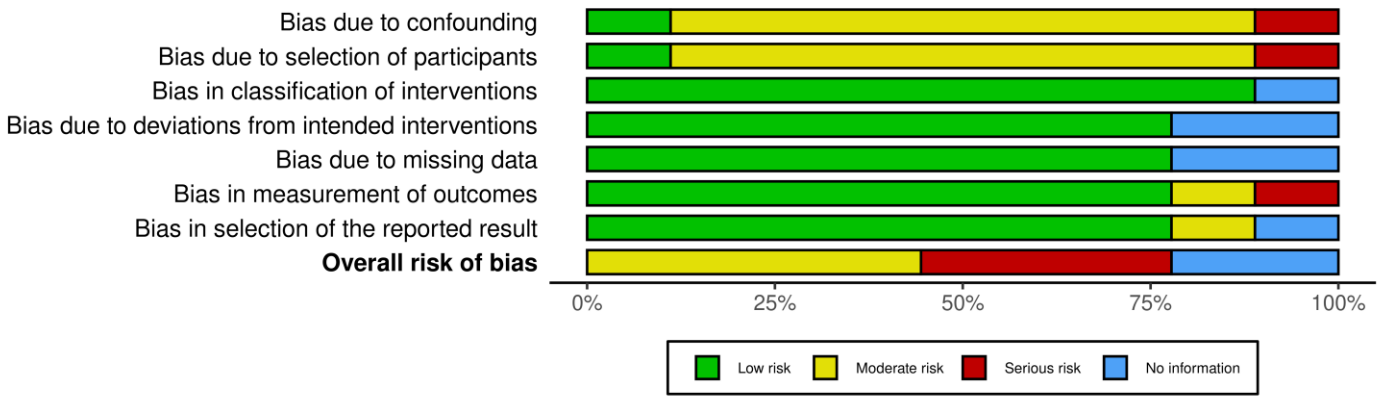

Supplementary Table S7. Robvis summary plot depicting the risk of bias in non-randomized included studies

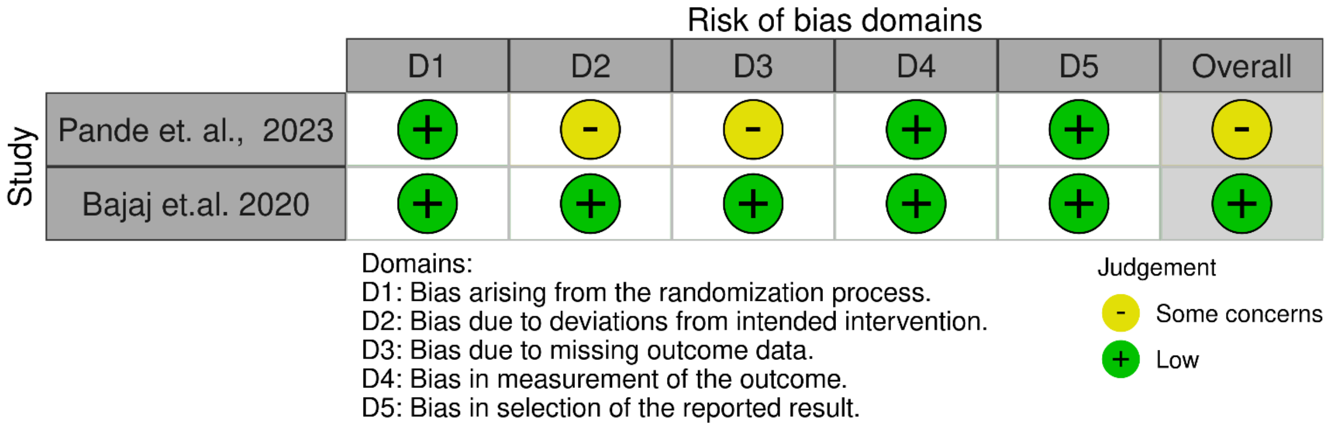

Supplementary Table S8. Robvis traffic light plot depicting RoB 2 in randomized controlled trials of included studies for survival outcomes

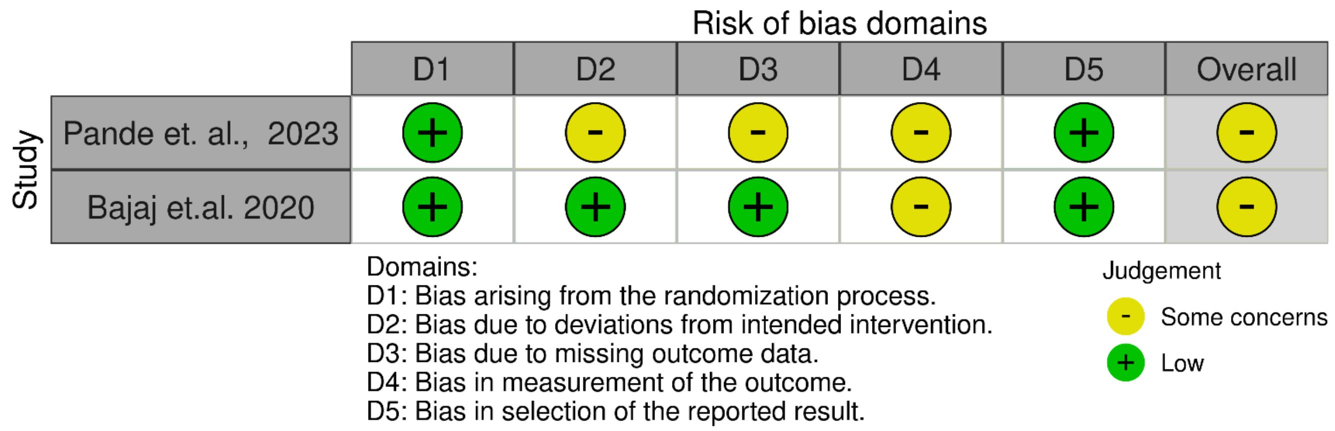

Supplementary Table S9. Robvis traffic light plot depicting RoB 2 in RCTs of included studies for abstinence after treatment

Certainty assessment							№ of patients		Effect		Certainty	Importance
№ of studies	Study design	Risk of bias	Inconsistency	Indirectness	Imprecision	Other considerations	FMT	SOC	Relative (95% CI)	Absolute (95% CI)		
**Overall survival (assessed with: Hazard ratio)**												
6	non-randomised studies	serious[a]	not serious	not serious	not serious	strong association	156 participants<br>-	156 participants<br>52.0%	**HR 0.50** (0.35 to 0.72) [Overall survival]	**21 fewer per 100** (from 29 fewer to 11 fewer)	⨁⨁◯◯ Low	CRITICAL
**Survival probability at 28 days**												
6	non-randomised studies	serious[a]	not serious	not serious	not serious	none	156 participants<br>95%	156 participants<br>79.0%	-	see comment<br>not pooled	⨁◯◯◯ Very low	
**Survival probability at 90 days**												
6	non-randomised studies	serious[a]	not serious	not serious	not serious	none	156 participants<br>80.0%	156 participants<br>64.0%	-	see comment<br>not pooled	⨁◯◯◯ Very low	
**Survival probability at 180 days**												
6	non-randomised studies	serious[a]	not serious	not serious	not serious	none	156 participants<br>80%	156 participants<br>62.0%	-	see comment<br>not pooled	⨁◯◯◯ Very low	
**Recurrence of alcoholism after treatment (assessed with: OR)**												
4	non-randomised studies	serious[a]	not serious	not serious	not serious	none	17/116<br>14.7%	30/107<br>28%	**OR 0.33** (0.05 to 2.00)	**166 fewer per 1 000** (from 261 fewer to 158 more)	⨁◯◯◯ Very low	

Supplementary Table S10. Summary of the quality of evidence by GRADEpro for survival outcomes and recurrence of alcoholism after treatment

CI: confidence interval; HR: hazard ratio; OR: odds ratio

Explanations:

a. Majority of the included studies presented notable concerns regarding the risk of bias. The elevated risk of bias primarily stemmed from participant selection and confounding factors, as the studies predominantly followed retrospective designs and did not clearly report how they managed confounders. Consequently, the authors decided to downgrade the level of evidence by one level.

References

1. Pandey, S. metaSurvival: Meta-analysis of a single survival curve using the multivariate methodology of DerSimonian and Laird. **2022**.
2. Rohatgi, A. *WebPlotDigitizer*, 5.2.
3. Egger, M.; Smith, G.D.; Schneider, M.; Minder, C. Bias in meta-analysis detected by a simple, graphical test. *BMJ* **1997**, *315*, 629–634, doi:10.1136/bmj.315.7109.629.
4. Harrer, M.; Cuijpers, P.; Furukawa, T.A.; Ebert, D.D. *Doing Meta-Analysis with R: A Hands-On Guide*, 1st ed.; Chapman and Hall/CRC: 2021.
5. Team, R.C. R: A Language and Environment for Statistical Computing. **2024**.
6. Balduzzi, S.; Rücker, G.; Schwarzer, G. How to perform a meta-analysis with R: a practical tutorial. *Evidence Based Mental Health* **2019**, *22*, 153–160, doi:10.1136/ebmental-2019-300117.
7. Schwarzer, G.; Carpenter, J.R.; Rücker, G. Meta-Analysis with R. *Use R!* **2015**, doi:10.1007/978-3-319-21416-0.
8. Harrer, M.; Cuijpers, P.; Furukawa, T.; Ebert, D.D. dmetar: Companion R Package For The Guide 'Doing Meta-Analysis in R'. **2019**.
9. Viechtbauer, W. Conducting meta-analyses in R with the metafor package. *Journal of Statistical Software* **2010**, *36*, 1–48, doi:10.18637/jss.v036.i03.
10. Therneau, T.M.; Grambsch, P.M. *Modeling Survival Data: Extending the Cox Model*; Springer: New York, 2000.
11. Therneau, T.M. A Package for Survival Analysis in R. **2024**.
12. Kassambara, A.; Kosinski, M.; Biecek, P. survminer: Drawing Survival Curves using 'ggplot2'. **2024**.
13. Therneau, T.M. coxme: Mixed Effects Cox Models. **2024**, doi:10.32614/CRAN.package.coxme.
14. Knapp, G.; Hartung, J. Improved tests for a random effects meta-regression with a single covariate. *Statistics in Medicine* **2003**, *22*, 2693–2710, doi:https://doi.org/10.1002/sim.1482.
15. IntHout, J.; Ioannidis, J.P.A.; Borm, G.F. The Hartung-Knapp-Sidik-Jonkman method for random effects meta-analysis is straightforward and considerably outperforms the standard DerSimonian-Laird method. *BMC Medical Research Methodology* **2014**, *14*, 25, doi:10.1186/1471-2288-14-25.
16. Jackson, D.; Law, M.; Rücker, G.; Schwarzer, G. The Hartung-Knapp modification for random-effects meta-analysis: A useful refinement but are there any residual concerns? *Statistics in Medicine* **2017**, *36*, 3923–3934, doi:https://doi.org/10.1002/sim.7411.
17. Veroniki, A.A.; Jackson, D.; Viechtbauer, W.; Bender, R.; Bowden, J.; Knapp, G.; Kuss, O.; Higgins, J.P.T.; Langan, D.; Salanti, G. Methods to estimate the between-study variance and its uncertainty in meta-analysis. *Research Synthesis Methods* **2016**, *7*, 55–79, doi:https://doi.org/10.1002/jrsm.1164.
18. Higgins, J.P.; Thompson, S.G. Quantifying heterogeneity in a meta-analysis. *Stat Med* **2002**, *21*, 1539–1558, doi:10.1002/sim.1186.
19. Kaplan, E.L.; Meier, P. Nonparametric Estimation from Incomplete Observations. *Journal of the American Statistical Association* **1958**, *53*, 457–481, doi:10.1080/01621459.1958.10501452.
20. Cheung, M.W. Modeling dependent effect sizes with three-level meta-analyses: a structural equation modeling approach. *Psychol Methods* **2014**, *19*, 211–229, doi:10.1037/a0032968.
21. Combescure, C.; Foucher, Y.; Jackson, D. Meta-analysis of single-arm survival studies: a distribution-free approach for estimating summary survival curves with random effects. *Statistics in Medicine* **2014**, *33*, 2521–2537, doi:https://doi.org/10.1002/sim.6111.
